# Functional and Character Disparity Are Decoupled in Turtle Mandibles

**DOI:** 10.1002/ece3.70557

**Published:** 2024-11-13

**Authors:** Jasper Ponstein, Guilherme Hermanson, Merlin W. Jansen, Johan Renaudie, Jörg Fröbisch, Serjoscha W. Evers

**Affiliations:** ^1^ Humboldt‐Universität zu Berlin Berlin Germany; ^2^ Museum für Naturkunde Berlin Berlin Germany; ^3^ Oertijdmuseum WB Boxtel Netherlands; ^4^ Department of Geosciences University of Fribourg Fribourg Switzerland

**Keywords:** biomechanics, disparity, form–function relationships, lower jaw, mandible, turtle

## Abstract

Turtles have high shape variation of their mandibles, likely reflecting adaptations to a broad variety of food items and ingestion strategies. Here, we compare functional disparity measured by biomechanical proxies and character disparity measured by discrete morphological characters. Functional and character disparities vary between clades and ecological groups and are thus decoupled. Comparisons with cranial disparity also indicate decoupled patterns within the turtle skull. Exploration of mandibular patterns reveals that several biomechanical configurations or character state combinations can lead to the same feeding type (i.e., convergence) or that high functional disparity can be achieved at a low exhaustion of character state combinations (e.g., cryptodires). Dietary specialists show larger functional disparity than generalists, but the phylogenetically widespread generalist ecology leads to high character disparity signals in the ecotype. Whereas character disparity generally shows high phylogenetic signal, functional disparity patterns correspond to dietary specializations, which may occur convergently across different groups. Despite this, individual functional measurements have overlapping ranges across ecogroups and do not always conform to biomechanical expectations. Jaw opening and closing biomechanical advantages model trade‐offs between force transmission and opening/closing speeds, and turtles show a variety of combinations of values that we try to synthesize into several “jaw types”. Closing mechanical advantage shows that turtles retain high levels of force transmission at the anterior jaw end compared with other groups (e.g., pseudosuchians). This can possibly be explained as an evolutionary adaptation to retain high bite forces at small head sizes.

## Graphical abstract

Here, we compare functional disparity measured by biomechanical proxies and character disparity measured by discrete morphological characters in turtle jaws. Exploration of mandibular patterns reveals that several biomechanical configurations or character state combinations can lead to the same feeding type (i.e., convergence) or that high functional disparity can be achieved at a low exhaustion of character state combinations (e.g., cryptodires). Jaw opening and closing biomechanical advantages model trade‐offs between force transmission and opening/closing speeds, and turtles show a variety of combinations of values that we try to synthesize into several “jaw types”.

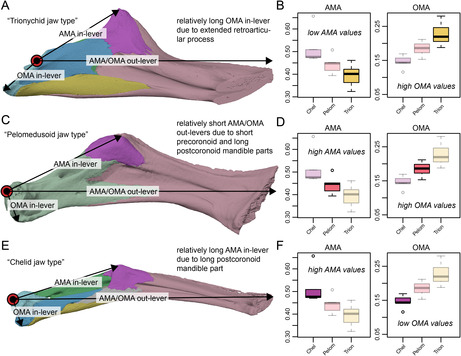

## 1 Introduction

Turtles (Testudinata) are an enigmatic clade of diapsid reptiles, and their crown‐group dates back to the Early Jurassic (Joyce et al. 2013; Pereira et al. 2017; Thomson, Spinks, and Shaffer 2021). The evolution of the unique turtle body plan, easily recognized by the presence of their shell and anapsid skull anatomy, has received considerable attention in the palaeontological and evolutionary biological literature (e.g., Schoch and Sues 2020; Lyson and Bever 2020). Despite the relatively low species richness of extant turtles (ca. 357 species recognized, TTWG 2021), the group occupies a wide range of habitats and dietary niches (Pritchard 1979; Ernst and Barbour 1989). Extant turtles live in marine, freshwater, and terrestrial habitats, and their fossil record documents multiple independent habitat transitions from land to water and vice versa (e.g., Yeh 1966; Claude et al. 2004; Gaffney, Tong, and Meylan 2006; Gaffney et al. 2011; Ferreira et al. 2015; Sterli 2015; Anquetin, Püntener, and Joyce 2017; Evers and Benson 2019; Joyce, Mäuser, and Evers 2021). Whereas most turtles feed either entirely in water or on land, some living species have the ability to feed both in aquatic environments as well as on land (e.g., Ernst and Barbour 1989; Summers et al. 1998; Natchev et al. 2009, 2015; Stayton 2011; Lemell et al. 2019). Extant and extinct turtles show dietary specializations such as herbivory, durophagy, or suction feeding, among others, which are commonly inferred by cranial or mandibular shape (Pritchard 1984; Claude et al. 2004; Bonin, Devaux, and Dupré 2006; Parham and Pyenson 2010; Foth, Rabi, and Joyce 2017; Joyce, Rollot, et al. 2021).

Due to its unique anatomy among extant amniotes, the turtle cranium has played a central role in studies on turtle ancestry and evolution (e.g., Gaffney 1979; Gaffney, Tong, and Meylan 2006; Joyce 2007; Sterli et al. 2010; Anquetin 2012; Bever et al. 2015; Schoch and Sues 2016; Joyce et al. 2016; Evers and Benson 2019; Werneburg and Maier 2019; Ferreira and Werneburg 2019). Cranial disparity of turtles is reasonably well understood, with various studies having assessed disparity based on linear measurements of functional significance (e.g., Herrel, O'Reilly, and Richmond 2002), geometric morphometrics (Claude et al. 2004; Ferreira et al. 2015; Foth and Joyce 2016; Foth, Rabi, and Joyce 2017; Foth, Ascurranz, and Joyce 2017; Hermanson et al. 2022), the topology of cranial bones (Miller et al. 2023), the topography of triturating surfaces (Shipps, Peecook, and Angielczyk 2023), or skull size (e.g., Claude et al. 2004; Hermanson et al. 2022). These studies variously focus on extant turtles or include fossils, and some of them study disparity trends through time, whereas most assess the relationships of cranial anatomy with ecology.

Whereas the turtle cranium is hypothesized to be influenced by many factors, such as ecology (e.g., Hermanson et al. 2022), the ability of turtles to retract their head into their shell (Werneburg 2015; Werneburg et al. 2015; Ferreira and Werneburg 2019; Ferreira et al. 2020; Hermanson et al. 2022), housing the central sense organs (e.g., Ferreira et al. 2023), allometry (e.g., Chatterji et al. 2022), ontogenetic constraints (e.g., Miller et al. 2023), among others, the mandible is a structure that is primarily adapted for feeding. Thus, disparity patterns, and leading on from that, ecological and functional influences on disparity, can potentially be measured more directly in the mandible than the cranium. Although turtle mandibles are known to be diverse in structure, with variation observed in terms of overall shape as well as in the constituent bones (Gaffney 1979; Evers et al. 2023), few studies have systematically documented anatomical variation between taxa (e.g., Gaffney 1979, 1982; Ferreira et al. 2015; Evers et al. 2023). Here, we assess the mandibular disparity of a large sample of extant turtles by two distinct methods. First, we measure functional disparity of turtle mandibles using linear measurements that signify traits commonly used to study jaw function in vertebrates (e.g., Anderson, Friedman, and Ruta 2013; Benevento, Benson, and Friedman 2019; Foffa, Young, and Brusatte 2024). Secondly, we compute disparity from a set of discrete characters originally designed by Evers et al. (2023) to contrast with the functional disparity.

## 2 Materials and Methods

### 2.1 Taxonomic Sample

The linear measurements and discrete characters were collected for the same set of specimens. For the collection of these data, we used 3D models of turtle mandibles from the dataset of Evers et al. (2023), which contained 70 extant turtle species covering the phylogenetic breadth of the group. Evers et al. (2023) used a poorly resolved CT scan of *Peltocephalus dumerilianus* (MCT RR 354). Here, we used a newly generated, better resolved CT scan of a different specimen, which we uploaded to MorphoSource (SMF 65407; https://www.morphosource.org/concern/media/000581038). Also, one of the specimens used in Evers et al. (2023) (NHMUK 76.1.31.19) was re‐identified as *Trachemys ornata* (previously misidentified as *Chrysemys picta* ), and thus relabelled as such in our File S1. We expanded the original dataset slightly by adding seven species. These additional species are the herbivorous emydid *Pseudemys concinna* , the carnivorous emydid *Emydoidea blandingii* , the herbivorous geoemydids *Hardella thurjii* , *Pangshura tecta* , *Heosemys annandalii* , and *Notochelys platynota*, as well as the omnivorous geoemydid *Orlitia borneensis* . These were also measured and scored based on 3D models that we downloaded from MorphoSource. This resulted in a total of 77 turtle species (21.5% of extant species diversity) and included at least one representative of each major subclade (“subfamily” level in Linnean taxonomy; see File S1).

### 2.2 Ecological Classifications

We classified each turtle into a set of ecological categories, for which we used the ecological data compiled in Hermanson et al. (2022). We used our ecological categories to color code species data in the descriptive statistics below, and all categorizations are provided in File S1. We used primary diet (carnivory, herbivory, and omnivory) for a gross categorization of turtles and specialized diet (suction feeding, durophagy, high‐fiber herbivory, and generalistic) to distinguish between important feeding modes that turtles display. These were chosen in part because they have either been hypothesized or shown to have influence on cranial shape (e.g., Claude et al. 2004; Ferreira et al. 2015; Hermanson et al. 2022; Shipps, Peecook, and Angielczyk 2023). In addition, we recorded the capacity of terrestrial feeding (yes, no) for all turtle species because feeding biomechanics may be influenced by under‐water or on‐land food processing (e.g., Stayton 2011; Herrel, Van Wassenbergh, and Aerts 2012; Heiss, Aerts, and Van Wassenbergh 2018; Lemell et al. 2019). Lastly, we also used habitat ecology (terrestrial, freshwater, and marine). In addition, our primary data file (File S1) also records the principal clade to which each taxon belongs. The ecological categories used herein are not independent of taxonomy/clade identity. For example, marine habits are only present among extant turtles in the subclade Chelonioidea, and most terrestrially feeding species are clustered in the obligate terrestrial subclade Testudinidae (tortoises), which encompass 13 out of 19 terrestrial turtles in our sample. This is unavoidable, as ecologies evolve along phylogenetic lineages and as not all ecologies have evolved convergently in different lineages among extant turtles. For this reason, we examine below (see Results and Discussion) if ecological disparity patterns can be explained solely by taxonomy instead.

### 2.3 Continuous Functional Measurements

**FIGURE 1:**
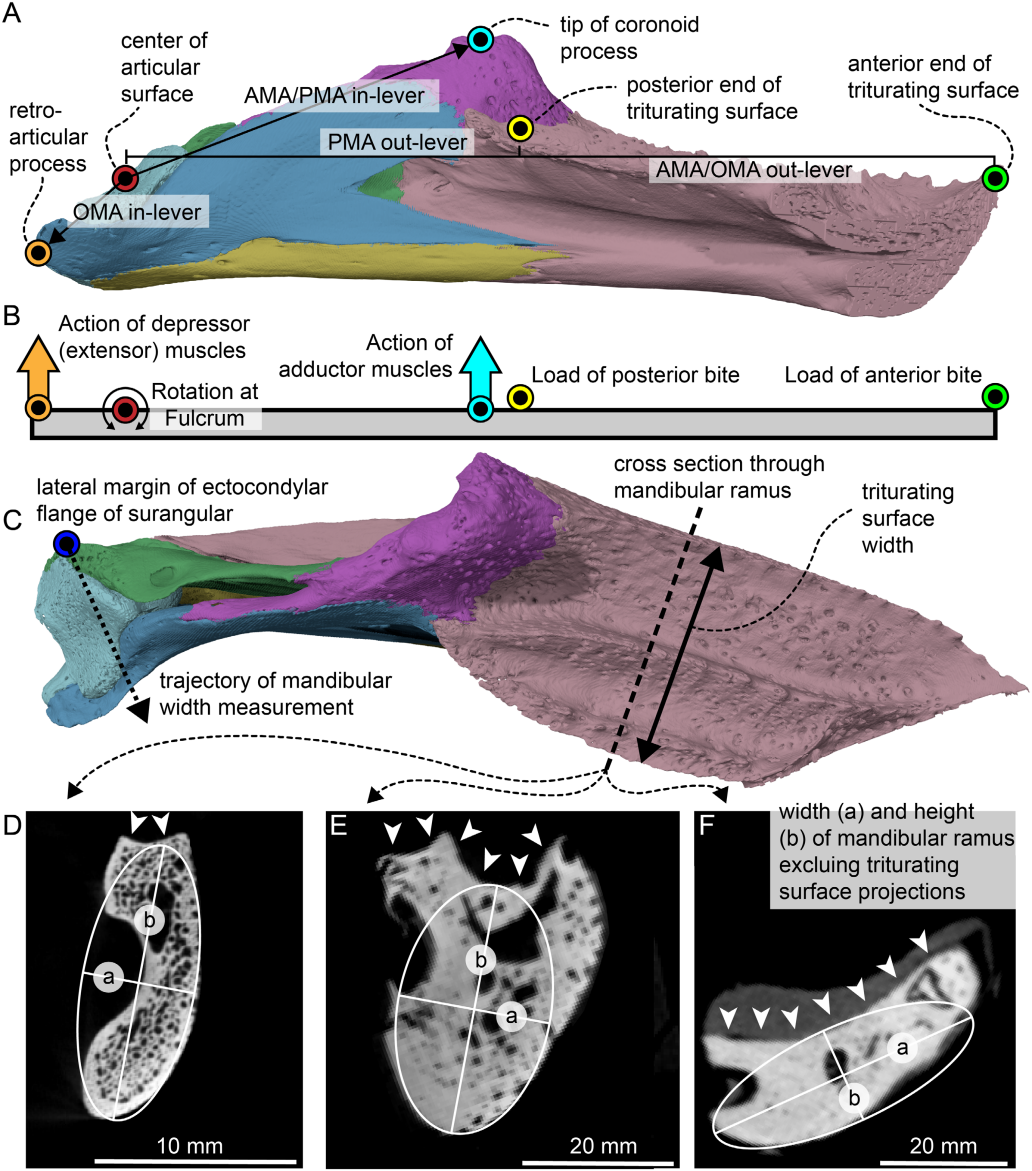
Schematic representation of the most important functional measurements. (A) Turtle mandible example in medial view to show lever‐measurements taken in the sagittal plane. Note that lever‐measurements are indicated by black lines or arrows, whereas colored circles represent the position of anatomical landmarks that define these measurements. (B) Schematic representation of turtle mandible as a lever. (C) Turtle mandible example in dorsal view to show triturating width measurement and plane for cross‐sectional images that underly the Second Moment of Inertia measurements. (D) Example cross‐section from a turtle with a high but narrow mandible ( *Eretmochelys imbricata* ). (E) Example cross‐section from a turtle with a very common morphology ( *Podocnemis unifilis* ). (F) Example cross‐section from a turtle with a very wide mandibular ramus ( *Graptemys geographica* ). White arrow heads in (D–F) indicate the dorsal surface of the bony triturating surface. AMA, anterior mechanical advantage; OMA, opening mechanical advantage; PMA, posterior mechanical advantage.

To study functional disparity in turtle mandibles, we used six biomechanically relevant measurements (Figure 1). These are inspired by previous biomechanical studies that focused on mandibles of various gnathostome lineages (Paleozoic fishes: Anderson 2009a; Anderson et al. 2011; early tetrapods: Anderson, Friedman, and Ruta 2013; teleosts: Westneat 2003; Mesozoic pseudosuchians: Stubbs et al. 2013; herbivorous dinosaurs: MacLaren et al. 2017; mammals: Benevento, Benson, and Friedman 2019; theropod dinosaurs: Ma et al. 2020; herbivorous Triassic tetrapods: Singh et al. 2021; and marine reptiles: Foffa, Young, and Brusatte 2024), but which have so far never been applied to turtles. As all living turtle taxa are edentulous, dental characters are not applicable and were not used. However, we added a new functional measurement related to the triturating surfaces of turtles, which has previously been noted to have functional relevance (Dalrymple 1977; Bever 2008, 2009; Shipps, Peecook, and Angielczyk 2023). In the following section, we describe each of our functional measurements and provide a justification for their use.

#### 2.3.1 Anterior, Posterior, and Opening Mechanical Advantage

Three continuous measurements are related to mechanical advantage, a commonly used measurement in biomechanical studies across gnathostomes (e.g., Maynard‐Smith and Savage 1959; Emerson 1985; Westneat 2003; Kammerer, Grande, and Westneat 2006; Stayton 2006; Rayfield et al. 2007; Anderson 2009a; Sakamoto 2010; Anderson et al. 2011; Anderson, Friedman, and Ruta 2013; Stubbs et al. 2013; Button, Rayfield, and Barrett 2014; Mallon and Anderson 2015; Nabavizadeh 2016; MacLaren et al. 2017; Navalón et al. 2019; Benevento, Benson, and Friedman 2019; Ma et al. 2020, 2022; Morales‐García et al. 2021; Singh et al. 2021, 2024; Meade and Ma 2022; Johnson et al. 2022; Schade et al. 2022; Foffa, Young, and Brusatte 2024). Mechanical advantage measurements are all based on the interpretation of the mandible as a lever, in which the mandible rotates around a fulcrum (i.e., the jaw joint) and has an input moment arm that characterizes the muscle insertion site with which movement is achieved and an output moment arm on which the movement is exerted (Westneat 1994, 2003; Figure 1). The relative position of the fulcrum to the input and output moment arms and the relative lengths of the latter determine the type of lever. In a third‐order lever, which is commonly applicable to gnathostome jaws (Stayton 2006; Anderson 2009a; Anderson et al. 2011; Anderson, Friedman, and Ruta 2013; Benevento, Benson, and Friedman 2019; Singh et al. 2021), the fulcrum is posterior to the moment arms and the input moment arm is shorter than the output moment arm. In a second‐order lever, the relative position of the fulcrum remains the same, but the output moment arm is shorter than the input moment arm. The ratio of input and output moment arms (i.e., in‐lever divided by out‐lever) generally determines the efficiency by which an input (=muscle) force is converted into an output force (=movement). Higher values of mechanical advantage signify a more powerful action at a lower amplification of motion (Westneat 1994, 2003), which is often equated with slower speed given that amplification of motion and speed are often correlated.

Thus, we used anterior and posterior mechanical advantage as measurements that characterize the relative jaw‐closing force and speed. In both, the in‐lever is defined as the distance between the fulcrum (i.e., jaw joint) and the point of muscle action. We used the center of the articular surface of the jaw joint as the starting point for all measurements that involve this structure (Figure 1). The point of muscle action is the anteriormost point of insertion site of the external adductor musculature, which in turtles can be placed at the coronoid process (e.g., Werneburg 2011, 2013; Ferreira and Werneburg 2019; Evers et al. 2023; Rollot et al. 2024). We used the tip of the coronoid process as a basis for respective measurements, as this point is easily identifiable even if the adductor musculature may extend anteriorly slightly beyond the tip of the coronoid process (e.g., Evers et al. 2023). The out‐lever in anterior mechanical advantage is the anteriormost tip of the jaw (Figure 1), which we hypothesize to be functionally equivalent to the first tooth position in toothed gnathostomes. The out‐lever in posterior mechanical advantage is the posterior end of the triturating surface (equivalent to the last tooth position; Figure 1). Thus, anterior mechanical advantage specifies an ‘anterior bite’ (resulting in a minimum possible value for mechanical advantage), while the posterior mechanical advantage specifies a ‘posterior bite’ (resulting in a maximum possible value for mechanical advantage). All distances were measured as straight lines along the sagittal plane (Figure 1).

The in‐lever may be larger than the out‐lever for posterior mechanical advantage in several turtles, for example when the posterior end of the triturating surface extends further posteromedially than the position of the coronoid process, such as the geoemydid *Batagur baska* (Evers et al. 2023). A similar condition in terms of a posterior dental extension is also found in the early synapsid *Edaphosaurus boanerges* (Modesto 1995) and some derived herbivorous ornithischians (i.e., hadrosaurids and ceratopsids; MacLaren et al. 2017). In these herbivorous species, posterior bites must be modeled as a second‐order lever, and posterior mechanical advantage values become greater than 1. We used posterior mechanical advantage regardless of whether a third‐ or second‐order lever is concerned. In a third‐order lever, mechanical advantage values are limited to take values from 0 to 1, whereby values approaching 1 signify increased bite force and reduced jaw‐closing speed. Values above 1, as realized in a second‐order lever, simply signify even greater increases of power and greater reductions of speed. Although both anterior and posterior mechanical advantages are related, they encode different aspects of mandibular biomechanics (Anderson 2009a) and vary independently in turtles (see Results), justifying their combined use.

We used jaw opening mechanical advantage (OMA) as a proxy for velocity of jaw opening (Westneat 1994). Jaw opening mechanics in gnathostomes can be modeled as a first‐order lever, in which the fulcrum (i.e., jaw joint) is positioned between the input and output moment arms (Westneat 1994). The input moment arm is the muscle insertion site for the jaw depressor muscles, which in turtles attach to the posteroventral end of the mandible, at the retroarticular process, but posterior to the position of the jaw joint (e.g., Werneburg 2011, 2013; Evers et al. 2023; Rollot et al. 2024). Thus, we characterized the depressor musculature attachment site by the distance between the jaw joint and the posteriormost point of the jaw (Figure 1). The output moment arm is the part of the mandible that is rotated around the fulcrum and can thus be measured as the distance between the jaw joint and the anteriormost point of the mandible (or anteriormost tooth position as a functional equivalent; Figure 1). All measurements were again taken in the sagittal plane. Jaw OMA was then calculated by dividing the input moment arm by the output moment arm, and low values correspond to fast jaw opening movement and vice versa (MacLaren et al. 2017; Ma et al. 2020).

#### 2.3.2 Second Mandibular Moment of Inertia (SMOI)

Stiffness of the mandible is an important mechanical property that describes the resistance of an object to deformation caused by bending (Wainwright et al. 1976). Stiffness quantifies how much force is required to deform an object by a unit distance. In functional studies, stiffness is usually approximated by a series of simplifications (e.g., Summers, Ketcham, and Rowe 2004; Anderson 2009a). Stiffness is proportional to the area moment of inertia (Wainwright et al. 1976), which is a geometrical property of a cross‐sectional area that describes the distribution of material around a neutral axis (Wainwright et al. 1976). In jaws, the neutral axis represents the anteroposterior axis of the mandibular ramus. The area moment of inertia can further be simplified by approximating the true cross‐sectional shape by an ellipse that captures the maximum height and width of the jaw ramus (Anderson 2009a). For an ellipse, area moment of inertia *I*_na_ is given by:

$$I_{\mathrm{na}}=\mathrm{\pi a}b^{3}4^{-1}$$

Whereby *a* represents the ellipse's axis length perpendicular to the applied force and *b* the axis length parallel to the applied force (Figure 1). In most turtle jaws, the mandibular ramus is higher dorsoventrally than it is mediolaterally wide, resulting in *a* being shorter than *b*. Typically, these values are measured at the peak bending moment, where the net adductor force acts (e.g., Anderson 2009a). However, this metric may overestimate resistance to bending if the jaw is lateromedially thin at its tallest point (Button, Rayfield, and Barrett 2014), as in trionychians, which have extremely high coronoid processes. In addition, the biting usually happens anterior to the coronoid process. As we were interested in measuring differences in stiffness of the mandibular ramus, which can be very robust or gracile (e.g., Evers et al. 2023), we opted to measure the ellipse at the midpoint of the dentary ramus. Hereby, we fit our ellipse to cross‐sectional CT slice images of the main ramus of the mandible. These images were generated by re‐slicing CT scans such that the mandibular ramus lies perpendicularly in the coronal plane. When fitting the ellipse using the ellipse measurement tool of the segmentation software Mimics v. 24 (http://biomedical.materialise.com/mimics), we excluded any dorsomedial expansions of the triturating surface, which sometimes form medially projecting plateaus that affect the width of the triturating surface (Figure 1; File S2: Figures S1–S5), which we have measured separately (see below). In the final step, we standardized this metric by comparing the area moment of inertia measurement of each species‐specific ellipse to that of a reference circle of the same area, following Summers, Ketcham, and Rowe (2004) and Anderson (Anderson 2009a). This makes the final SMOI value dimensionless rather than a distance to the fourth power.

The area moment of inertia for a circle is given by:

$$I_{c}=\pi r^{4}4^{-1}=\pi {\left(\mathrm{ab}\right)}^{2}4^{-1}$$

The SMOI for the species‐specific ellipse at the coronoid process can then easily be calculated by:

$$I_{\text{Second}}=\frac{I_{\mathrm{na}}}{I_{c}}=\frac{\mathrm{\pi a}b^{3}4^{-1}}{\pi {\left(\mathrm{ab}\right)}^{2}4^{-1}}=\frac{b}{a}$$

The greater the dorsoventral height relative to the mediolateral width of the mandibular ramus, the higher the SMOI. As stiffness is proportional to area moment of inertia, higher SMOI values indicate higher stiffness (i.e., less bending per applied force). Gnathostomes that exhibit high bite force or orally process tougher food items are expected to have mandibles with a higher stiffness (i.e., higher resistance to bending).

#### 2.3.3 Mandibular Length–Width Ratio

Absolute length of the mandible is a useful proxy for gape size and has been included as a continuous character in various biomechanical studies (Anderson 2009a; Anderson et al. 2011; Stubbs et al. 2013). However, for many turtle specimens from museums, exact ontogenetic ages are not known, making it impossible to use specimens of the same age across our entire sample. Although we used adult morphologies only for our sample, various specimens are not at the maximum size range for their species (e.g., chelonioids), possibly introducing taxon‐specific biases. Thus, we standardized jaw length by width. This aspect ratio also conveys important functional information: For example, previous studies have shown that two types of cranial shape (long but narrow and short but wide) are associated with suction feeding (e.g., Joyce, Rollot, et al. 2021; Hermanson et al. 2022). We measured mandibular length as the straight distance between the anterior tip of the jaw and the posterior tip of the mandible (usually the retroarticular process) along the sagittal plane. Jaw width was measured as the maximum width between the mandibular rami, which can be best measured as the straight distance between the lateral margins of the ectocondylar flanges of the right and left surangulars (Figure 1C).

#### 2.3.4 Relative Width of the Triturating Surface

The triturating surface is a key anatomical feature in the turtle skull, related to food processing (e.g., Gaffney 1979; Shipps, Peecook, and Angielczyk 2023), and was thus herein used as a separate continuous character. The trait has been measured in comparative studies on monospecific turtle studies (Dalrymple 1977; Bever 2008, 2009). We measured the maximum width of the triturating surface for any species. As triturating surface shape has significant variation across turtles (e.g., Evers et al. 2023), this means that the measurement was not taken at the same level of the mandible across turtles. For some species, the triturating surfaces are widest at the symphysis (e.g., chelonioids), whereas for other turtles the greatest triturating width is achieved somewhere along the dentary ramus (e.g., geoemydids). We measured the maximum triturating surface width in dorsal view (Figure 1) and then divided this value by the absolute length of the mandible to obtain a relative width measurement.

### 2.4 Functional Hypotheses

Our functional measurements allowed us to propose a priori expectations about the distribution of values for turtles, depending on their known feeding modes. These hypotheses can also be modeled after other gnathostomes, as the lever mechanics between different animals are the same. Mandibles of herbivores typically have higher anterior and posterior mechanical advantage than carnivores (Maynard‐Smith and Savage 1959; Stayton 2006; Navalón et al. 2019), as jaw‐closing speed is not relevant to herbivores and a stronger bite is helpful in cutting through tougher vegetation. Thus, we expect herbivorous turtles to conform to this expectation. In addition, we hypothesize that higher mechanical advantages are also seen in durophagous turtles. Turtles that actively pursue elusive prey are required to have faster bites, resulting in the expectation of relatively lower mechanical advantages. OMA is our proxy for jaw opening speed. Rapid jaw opening is more suitable for carnivores that actively pursue elusive prey (e.g., Hanken and Hall 1993; Emerson 1985; Kammerer, Grande, and Westneat 2006) and is thus expected to be an important trait for gape‐suction feeding specialists (e.g., Pritchard 1984; Van Damme and Aerts 1997; Aerts, van Damme, and Herrel 2001; Lemell et al. 2002, 2010, 2019; Bels et al. 2008; Joyce, Rollot, et al. 2021). In addition, herbivores are expected to have higher jaw OMA values, signifying lower speeds than carnivorous relatives (Ma et al. 2020). Our SMOI measurements signify resistance to bending stress. Gnathostomes that exhibit high bite force or orally process tougher food items are expected to have mandibles with a higher resistance to bending. Applied to turtles, we hypothesize that durophagous and high‐fiber herbivores have higher SMOI values than gape‐suction feeders. For the mandibular aspect ratio, we expect carnivorous non‐durophagous turtles to have relatively longer jaws and durophagous turtles to have relatively shorter but broader jaws. In addition, we expect high‐fiber herbivores to have broad but short jaws. Regarding the relative triturating surface width, we expect durophagous turtles to have relatively wider triturating surfaces than non‐durophagous turtles (e.g., Ferreira et al. 2015). In addition, turtles without significant mandibular food processing, such as gape‐suction feeders, are expected to have reduced triturating surfaces (e.g., Lemell et al. 2010; Joyce, Rollot, et al. 2021).

### 2.5 Discrete Character Data

We took the character matrix from Evers et al. (2023), encompassing 51 discrete characters related to the lower jaw, and expanded this with the scores of the seven species added to the original dataset (File S3). For three characters that were polymorphic for some species in the original matrix (characters 26, 27, and 39), we adopted a ‘majority’ scoring concept in which the most common condition was set as the character state (e.g., Garbin, Ascurranz, and Joyce 2018).

### 2.6 Data Ordination and Statistical Tests

We read our raw measurement data (File S1) into the statistical computing environment R (R Core Team 2021). We plotted the distributions of functional measurement values for turtles in boxplots (Tukey 1977), whereby turtles were grouped according to clade identity and ecology. We tested if group means are statistically significantly different by applying Bonferroni‐corrected pairwise *t*‐tests, using the pairwise *t*‐test function of the stats package (R Core Team 2021). Continuous data were subjected to principal component analysis (PCA) using the prcomp function of the stats package (R Core Team 2021). To do this, we standardized all continuous measurements by *Z*‐transformation so that the average value for each trait approaches 0 and the standard deviation approaches 1, ensuring equal weights are given to the individual measurement in the PCA (Benevento, Benson, and Friedman 2019). As a disparity metric for the continuous data, we calculated the sum of variances for ecological groups of interest from the PCA coordinates, using the dispRity function of the dispRity package (Guillerme 2018) and all PC axes. Sum of variances characterizes the volume that a subgroup occupies in morphospace (Guillerme et al. 2020). This disparity metric is relatively insensitive to small sample size effects (Wills 1998; Brusatte et al. 2008; Hopkins and Gerber 2017).

For the discrete character ordination, we computed a distance matrix from the character data with the daisy command of the cluster package (Maechler et al. 2023). The principal coordinate analysis (PCoA) was computed from this using the pcoa command from the ape package (Paradis and Schliep 2019). We then again calculated the sum of variance as a disparity metric for various ecological or taxonomic subgroups using all PCoA coordinates.

For both the continuous (=functional) and discrete (=character) disparity metrics, we performed a series of hypothesis tests that generally ask if one group of interest occupies a significantly larger volume of the ordination space than another. The four principal questions tested are described in Table 1. For hypothesis testing, we bootstrapped the disparity values to get distributions using the boot.matrix function of the dispRity package (Guillerme 2018). We then applied two‐sided pairwise Wilcoxon tests (i.e., Mann–Whitney test) via the test.dispRity function of the dispRity package (Guillerme 2018) using default settings, asking if two groups have significantly different distributions. We adjusted *p* values with a Bonferroni correction as implemented in the test.dispRity function via the *p*.adjust from the stats package (R Core Team 2021). All R code is provided in Zenodo (10.5281/zenodo.13938962) via GitHub (https://github.com/G‐Hermanson/Turtle‐jaw‐disparity).

**TABLE 1:** Disparity‐related questions we tested using both continuous and discrete datasets.

Disparity question tested
Do cryptodires and pleurodires have significantly different disparities?
Do turtles with different primary diets occupy significantly different volumes in morphospace?
Do turtles that have the capacity to feed on land occupy a significantly different volume in morphospace from turtles that cannot?
Do dietary specialists (e.g., durophages, suction feeders, high‐fiber herbivores) occupy significantly smaller volumes in morphospace than generalists?

## 3 Results

### 3.1 Functional Measurement Distributions Across Taxonomy and Ecology

**FIGURE 2:**
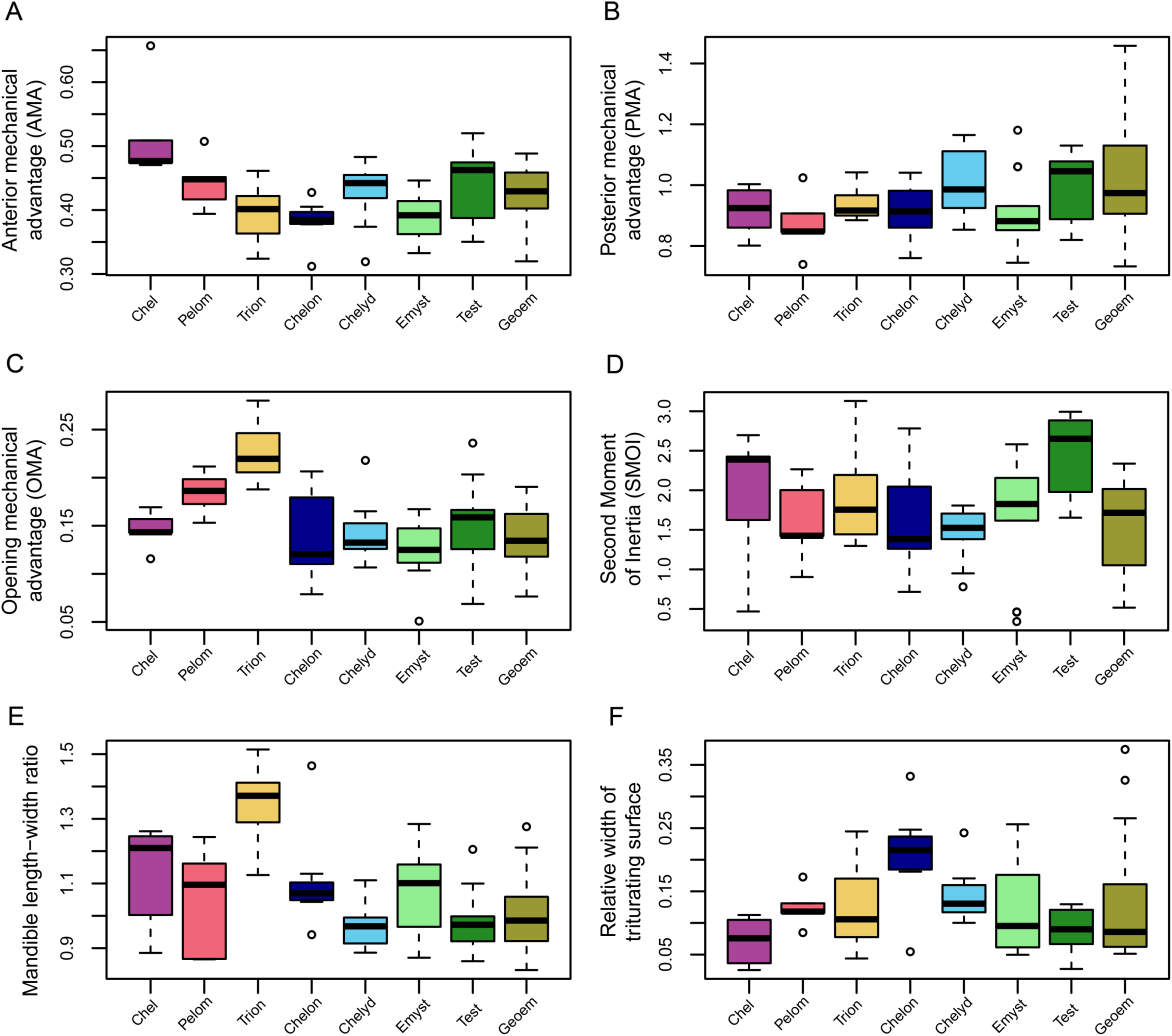
Boxplots of functional measurements for major turtle clades. Black bar within boxplots represents the median values, and boxes themselves indicate the interquartile range. Clade abbreviations: Chel, Chelidae; Chelon, Chelonioidea; Chelyd, Chelydroidea; Emyst, Emysternia; Geoem, Geoemydidae; Pelom, Pelomedusoides, Test, Testudinidae; Trion, Trionychia.

**FIGURE 3:**
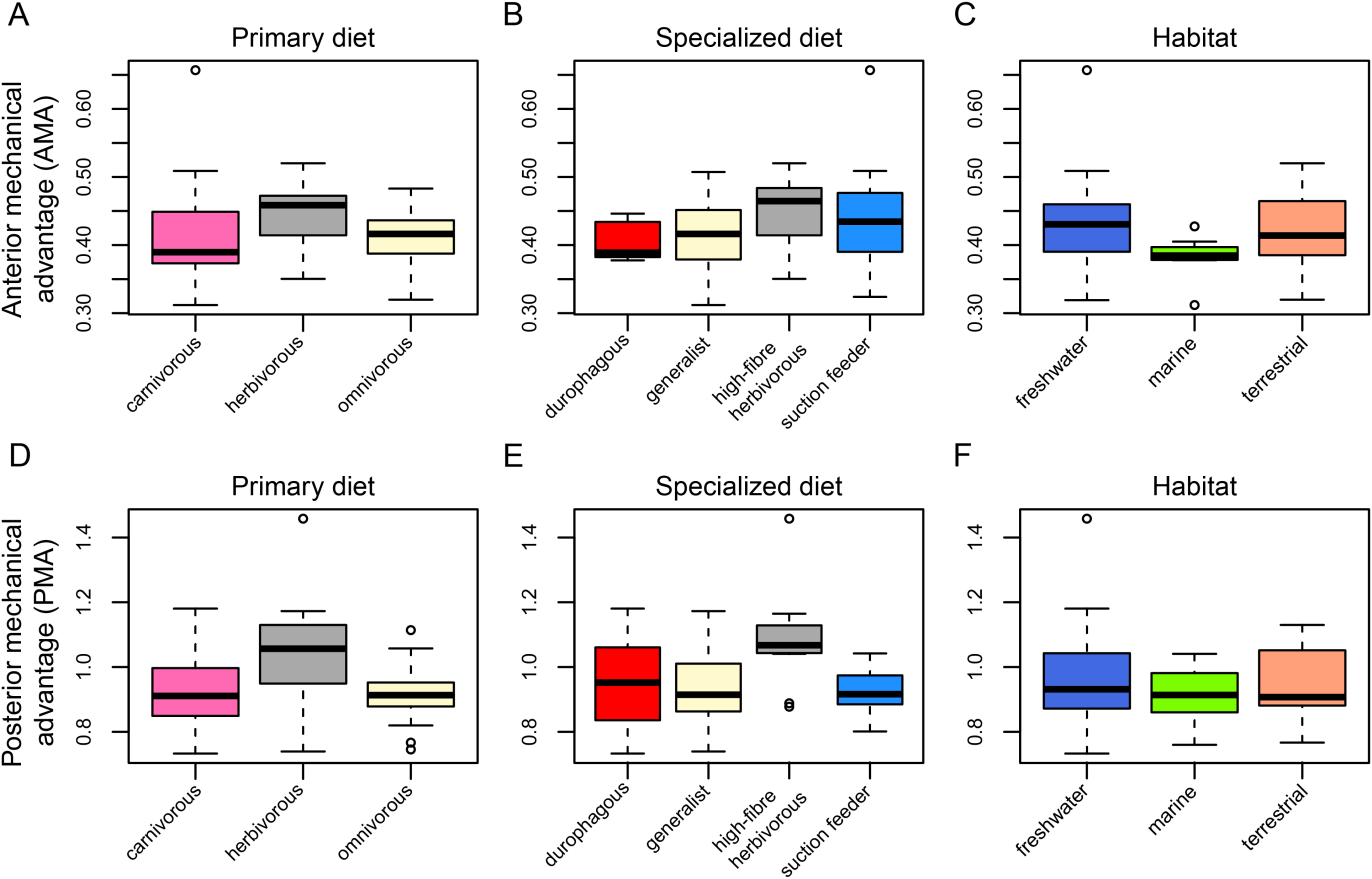
Boxplots of anterior and posterior mechanical advantage for ecological classifications of turtles. Black bar within boxplots represents the median values, and boxes themselves indicate the interquartile range.

Anterior mechanical advantage (AMA) values range from 0.3 ( *Dermochelys coriacea* ) to 0.66 (*Chelus fimbriatus*), although the majority of turtles has values around the global mean of 0.4 with a narrow standard deviation (SD = 0.05; Figure 2A). Most turtle clades have overlapping ranges for AMA, but chelids have significantly larger values than all other turtle clades (*p* < 0.05 for all pairwise comparisons; File S4; Figure 2A). Posterior mechanical advantage (PMA) values range from 0.73 ( *Malayemys subtrijuga* ) to 1.46 ( *Morenia ocellata* ), but again the distribution of the majority of turtles is narrow with a mean of 0.95 and SD = 0.13 (Figure 2B). These ranges indicate that turtles achieve a good (i.e., near one‐to‐one) force transmission at the posterior end of the jaw, whereas the force transmission is a lot lower at anterior biting points. Although AMA and PMA are significantly correlated (*p* = 6.7 × 10^−5^), the correlation is not strong (*R* = 0.44; File S2: Figure S6). This is primarily caused by pleurodires, in which both chelids and pelomedusoids have proportionally larger AMA values than cryptodires (Figure 2A,B), which can anatomically be explained by their long postcoronoid‐jaw length and relatively short dentary ramus that causes relatively long in‐levers (for both PMA and AMA) but a relatively short AMA out‐lever. When comparing AMA values among ecological groups, no pairwise comparison returns statistically different means (File S4; Figure 3A). Although high‐fiber herbivores have the largest mean among the specialist dietary categorizations, this does not translate to significant differences (Figure 3B; File S4), and habitat ecology groups are also not significantly different from one another (Figure 3C; File S4). Trends for posterior mechanical advantage are slightly different (Figure 3D–F), whereby herbivorous turtles have significantly higher mean PMA values than both carnivorous (*p* = 0.0005; File S4) and omnivorous turtles (*p* = 0.001; File S4) indicating better force transmission at lower closing speeds, as expected. This is reflected in the specialized diets, in which herbivores are again significantly higher in PMA values than generalists (*p* = 0.001) and suction feeders (*p* = 0.013), but only marginally non‐significantly different from durophagous turtles (*p* = 0.064; Figure 3E; File S4).

**FIGURE 4:**
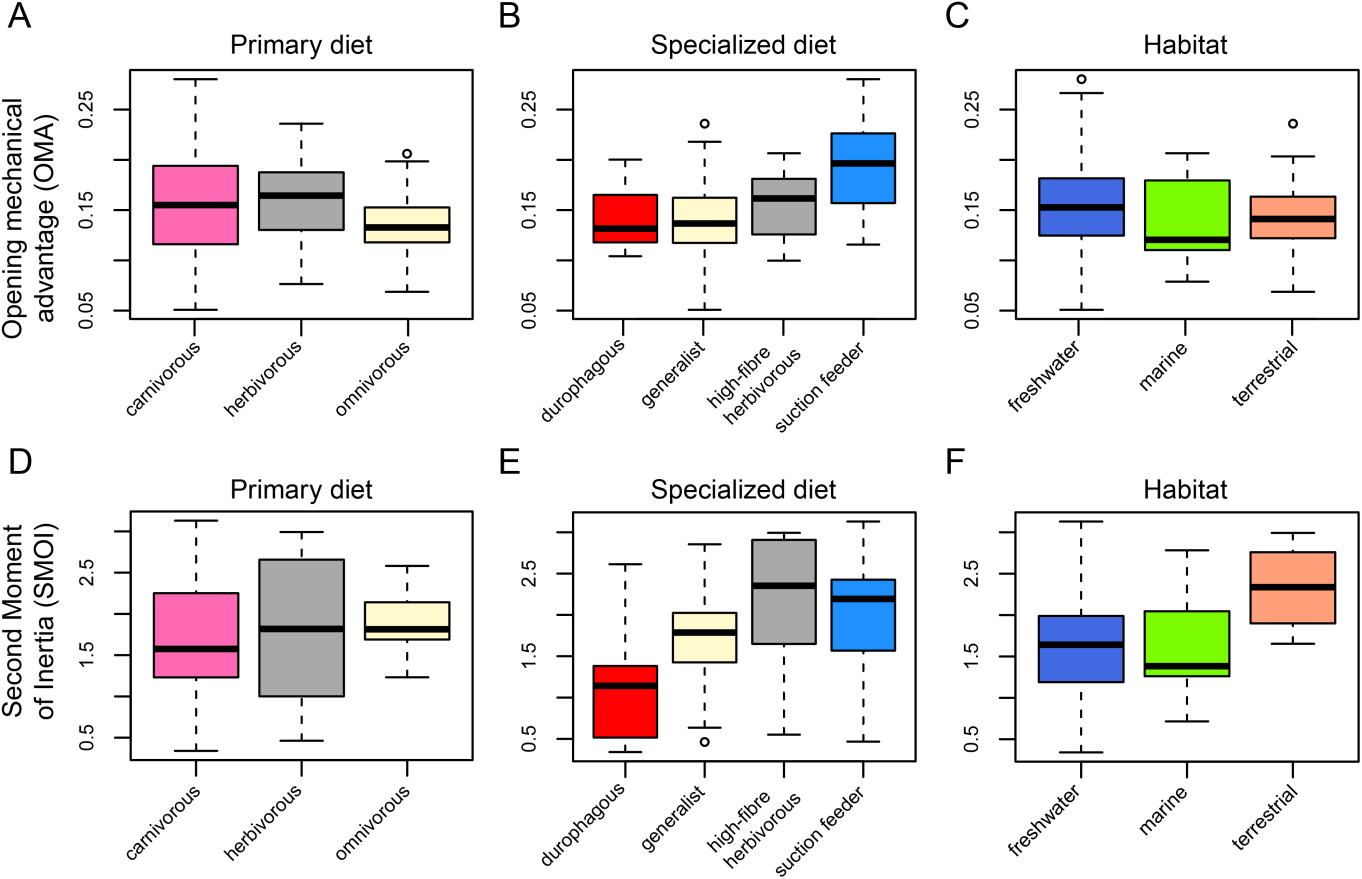
Boxplots of opening mechanical advantage and Second Moment of Inertia for ecological classifications of turtles. Black bar within boxplots represents the median values, and boxes themselves indicate the interquartile range.

OMA values range from 0.05 ( *Emydoidea blandingii* ) to 0.28 ( *Chitra chitra* ). Unlike with AMA and PMA, the general data distribution has a relatively larger variation of group means at a global mean of 0.15 ± 0.04. Hereby, trionychians have significantly larger OMA values than all other turtle subclades (all pairwise *p*‐values < 0.05; File S4; Figure 2C), indicating better force transmission at lower speeds compared to other turtles. This can anatomically be explained by the long retroarticular processes of trionychians, which cause the in‐lever to be long. Trionychians have statistically larger OMA values than all other groups (Figure 2C; File S4), which have overlapping distributions. Concerning primary dietary categories, omnivorous taxa show a distribution toward lower OMA values, although non‐significant (*p*_carnivorous_ = 0.1; *p*_herbivorous_ = 0.07; File S4; Figure 4A). Among the specialized diets, suction feeders have significantly higher OMA values than most other groups (*p*_durophagous_ = 0.02; *p*_generalist_ = 0.0005; *p*_herbivorous_ = 0.12; File S4; Figure 4B), but the taxonomic plots show that this is caused only by trionychids (Figure 2C). High‐fiber herbivores have elevated values in comparison to durophages and generalists (Figure 4B), but this is not statistically significant (File S4). Habitat ecology again shows no statistically significant differences between categories (File S4; Figure 4C).

SMOI values range from 0.34 ( *Graptemys geographica* ) to 3.13 ( *Cycloderma frenatum* ). Values below 1 describe jaws in which the major beam of the mandibular ramus is transversely wider than dorsoventrally high, whereas values above 1 indicate jaws that are mediolaterally thin and dorsoventrally high. The global mean and SD are 1.76 ± 0.69. Group‐specific means vary relatively strongly, as well as intra‐group variation. For example, chelydroids have narrow ranges around their mean values (chelydroids = 1.45 ± 0.36; File S1), whereas some other groups have larger standard deviations (chelonioids = 1.64 ± 0.76; geoemydids = 1.49 ± 0.62; chelids = 1.92 ± 0.9; File S1; Figure 2D). Among the clade‐wise comparisons, the only statistically significant differences regard testudinids, which have significantly larger SMOI values than chelydroids (*p* = 0.017) and geoemydids (*p* = 0.003; File S4; Figure 2D). When looking at ecological categorizations, terrestrial taxa have significantly higher values than marine or freshwater turtles (File S4; Figure 4F), but this is caused by the terrestrial category being mostly filled with testudinids, which have high SMOI values (Figure 2D). For dietary ecologies, durophages have significantly lower SMOI values than high‐fiber herbivores (*p* = 0.002) and suction feeders (*p* = 0.02), and high‐fiber herbivores only have significantly higher SMOI values than durophages (Figure 4F), whereas all the remaining pairwise comparisons remain statistically indistinct between categories (File S4).

**FIGURE 5:**
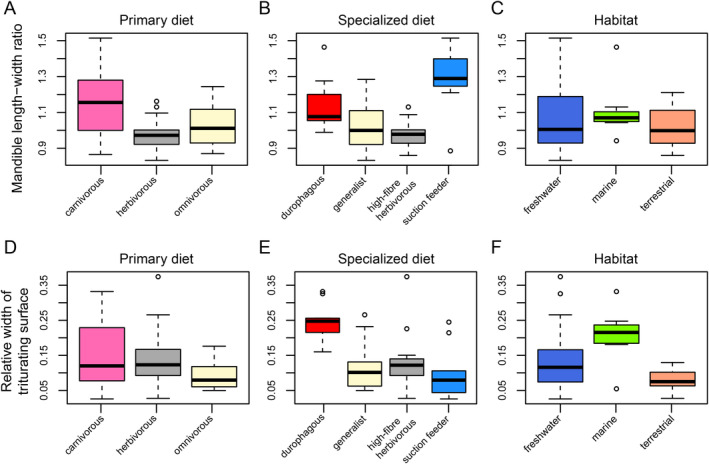
Boxplots of mandibular length–width ratio and relative triturating surface width for ecological classifications of turtles. Black bar within boxplots represents the median values, and boxes themselves indicate the interquartile range.

Turtles show a wide range of jaw aspect ratios, including taxa in which the jaw is wider than long as well as the opposite. The minimum length width ratio is 0.83 ( *Notochelys platynota* ) and the maximum is 1.51 ( *Chitra chitra* ), with the mean value indicating a mean shape of roughly equal length and width (mean = 1.06 ± 0.16). Trionychians are noteworthy as having statistically larger jaw aspect ratios than most other clades (pairwise *p*‐values < 0.05; File S4; Figure 2E), except for chelids. Although the interquartile range is narrow for some clades such as chelonioids or testudinids, these clades include strong outliers toward high aspect ratios (e.g., *Eretmochelys imbricata* ; *Kinixys erosa* ; Figure 2E). In terms of ecological classifications, carnivorous turtles have statistically significantly longer jaws (*p*_herbivorous_ = 2.05 × 10^−5^; *p*_omnivorous_ = 0.002; File S4; Figure 5A). This is caused by both durophagous and suction feeding turtles having statistically higher length–width ratios (all comparisons with other specialized ecologies *p* < 0.05; File S4; Figure 5B), whereby the suction feeders have even significantly larger values than the durophages (*p* = 0.04; File S4; Figure 5B). The taxonomic plots show that the high values of suction feeders, although primarily caused by trionychids, are also apparent in chelids as the second clade with a dominant subset of suction feeders (Figure 2E). Herbivores and high‐fiber herbivores among the ecological specializations have the lowest length–width ratios, with medians around 1, indicating the jaws are as broad as they are long (Figure 5A,B). Habitat ecologies are not different in their aspect ratio distributions (Figure 5C).

Relative triturating surface widths range from 0.02 (*Chelus fimbriatus*) to 0.37 ( *Morenia ocellata* ), whereby the global mean value is 0.13 ± 0.07. Chelonioids have a large range of values due to the narrow triturating surface of *Dermochelys coriacea* , which equals to 5% of the jaw length. However, even including *Dermochelys coriacea* , chelonioids have statistically significantly larger values than most other turtles, with the exception of chelydroids and pelomedusoids (File S4; Figure 2F). Chelids have the lowest mean values (mean = 0.07 ± 0.04), followed by testudinids (mean = 0.09 ± 0.03). Omnivorous taxa have significantly narrower relative triturating surfaces than carnivorous turtles (*p* = 0.01), but only marginally narrower than herbivorous species (*p* = 0.057; File S4; Figure 5D). However, this does not translate to statistically significantly high values in high‐fiber herbivores compared to generalists (File S4; Figure 5E). The specialized ecological categorization furthermore reveals that the high values for carnivorous turtles are entirely driven by durophages, which have significantly higher values than all other categories (all *p* values < 0.05; File S4; Figure 5E). Suction feeders, as expected, have the lowest values, but these are not significantly smaller than generalists or high‐fiber herbivores. All habitat ecology classes are statistically different to one another (File S4; Figure 5F), but this is partially because the taxonomic groups closely match certain ecologies (Figure 2F), with all marine taxa being chelonioids and many terrestrial taxa being testudinids.

### 3.2 Principal Component Analysis of Functional Measurements

**FIGURE 6:**
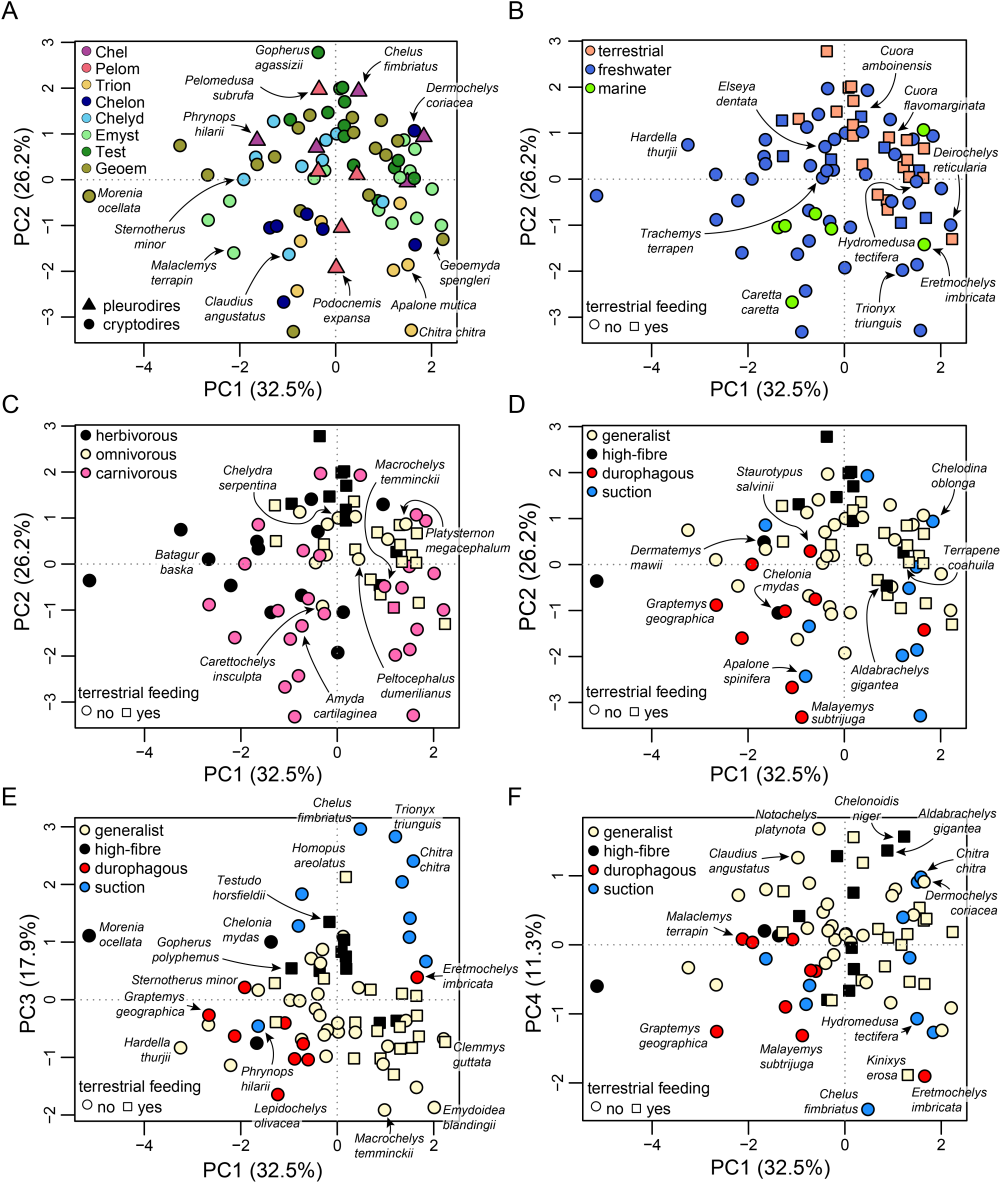
PCA morphospace of functional jaw measurements based on first four principal components, with various grouping superimposed on the data. (A) Major turtle clades along PC1 and PC2. (B) Habitat ecology along PC1 and PC2. (C) Primary diets along PC1 and PC2. (D) Specialized diets along PC1 and PC2. (E) Specialized diets along PC1 and PC3. (F) Specialized diets along PC1 and PC4. See Figures S10–S12 in File S2 for repeated plots in which all species are indicated by numbers. Note the different color and point‐symbol keys per panel. Clade abbreviations: Chel, Chelidae; Chelon, Chelonioidea; Chelyd, Chelydroidea; Emyst, Emysternia; Geoem, Geoemydidae; Pelom, Pelomedusoides, Test, Testudinidae; Trion, Trionychia.

Ordination of functional jaw measurements of turtles into a principal component morphospace reveals limited patterns of functional disparity that could be visually extracted from the data distribution (Figure 6). The first two principal components collectively explain 58.7% of the total variance (Figure 6, Table 2). The third principal component explains 17.9% of the total variance, whereas the fourth axis explains 11.3% of the total variance. Smaller PCs (Table 2) are not shown in our main figures. The eigenvector coefficients of the PCA table reveal which functional traits are important in explaining the individual principal components (Table 2). PC1 (32.5% of total variance) distinguishes between mandibles with a relatively narrow triturating surface and low anterior, posterior, and OMAs, relatively elongate‐but‐narrow mandibles with a high‐oval cross‐section (resulting in high SMOI) at high PC1 values, like *Eretmochelys imbricata, Deirochelys reticularia*, or *Geoemyda spengleri* . Low PC1 values are found in turtles with opposite traits, such as *Morenia ocellata* . However, there is no taxonomic or ecological separation of groups along PC1, with some clades spanning the entire range of possible values (e.g., geoemydids), and other clades with more restrictive ranges (e.g., chelids, trionychians), nevertheless overlapping with all other groups (Figure 1). PC2 (26.2% of total variance) describes relatively short mandibles with narrow triturating surfaces and fast jaw opening at high PC2 values, like testudinids, and relatively elongate mandibles with broad triturating surfaces and slow jaw opening speed at low values, like trionychians or the durophagous geoemydid *Malayemys subtrijuga* (Figure 2). PC2 has a somewhat clearer group distinction than PC1, with terrestrial feeders and terrestrial turtles being largely confined to positive values and marine and durophagous turtles being largely confined to negative PC2 values. For marine turtles, *Dermochelys coriacea* is a notable exception at very high PC2 values. Principal diets are visually not well separated in the morphospace (Figure 6). Nevertheless, we base our disparity interpretation on statistical tests of comparisons between the sum of variances for our various groupings, and these are given below. PC3 (17.9% of total variance) describes mandibles with high OMA at high PC3 values. These are particularly pronounced in suction feeding turtles (Figure 6E), such as *Chelus fimbriatus* or trionychids. Low PC3 values correspond to mandibles with low SMOI values, such as in *Lepidochelys olivacea* , *Hardella thurjii* , or *Macrochelys temminckii* . The specialized diets show the best partial separation of groups in the morphospace along PC3 (Figure 6E), whereby suction feeders generally plot at higher values than generalists or durophages, with some exceptions (e.g., *Phrynops hilarii* , *Eretmochelys imbricata* ). PC4 (11.3% of total variance) describes mandibles with low AMA and low mandible length–width ratios paired with high OMA at high PC4 values, as in *Notochelys platynota* , *Chelonoidis niger*, or *Aldabrachelys gigantea* (Figure 6F). Species at low PC4 values have mandibles with opposite trend, such as *Chelus fimbriatus*, *Eretmochelys imbricata* , and *Kinixys erosa* (Figure 6F). Ecological or taxonomic group separation is poor along PC4, although high‐fiber herbivores have a slight tendency for higher PC4 values and durophages for lower PC4 values (Figure 6F).

**TABLE 2:** Results of PCA using biomechanical measurements of turtle mandibles.

	PC1	PC2	PC3	PC4	PC5	PC6
Eigenvalues	1.39	1.25	1.03	0.82	0.68	0.5
Proportion of variation explained (%)	32.5	26.2	17.9	11.3	7.9	4.2
Eigenvector coefficients						
Anterior mechanical advantage (AMA)	−0.278	0.479	0.413	−0.524	0.493	0.045
Posterior mechanical advantage (PMA)	−0.519	0.338	0.224	0.129	−0.638	−0.374
Opening mechanical advantage (OMA)	−0.147	−0.356	0.699	0.543	0.249	0.063
Second moment of inertia (SMOI)	0.494	0.382	0.356	0.043	−0.398	0.567
Mandible length–width ratio	0.261	−0.505	0.390	−0.594	−0.292	−0.292
Relative width of triturating surface	−0.563	−0.355	−0.101	−0.240	−0.205	0.667

### 3.3 Principal Coordinate Analysis of Discrete Character Matrix

**FIGURE 7:**
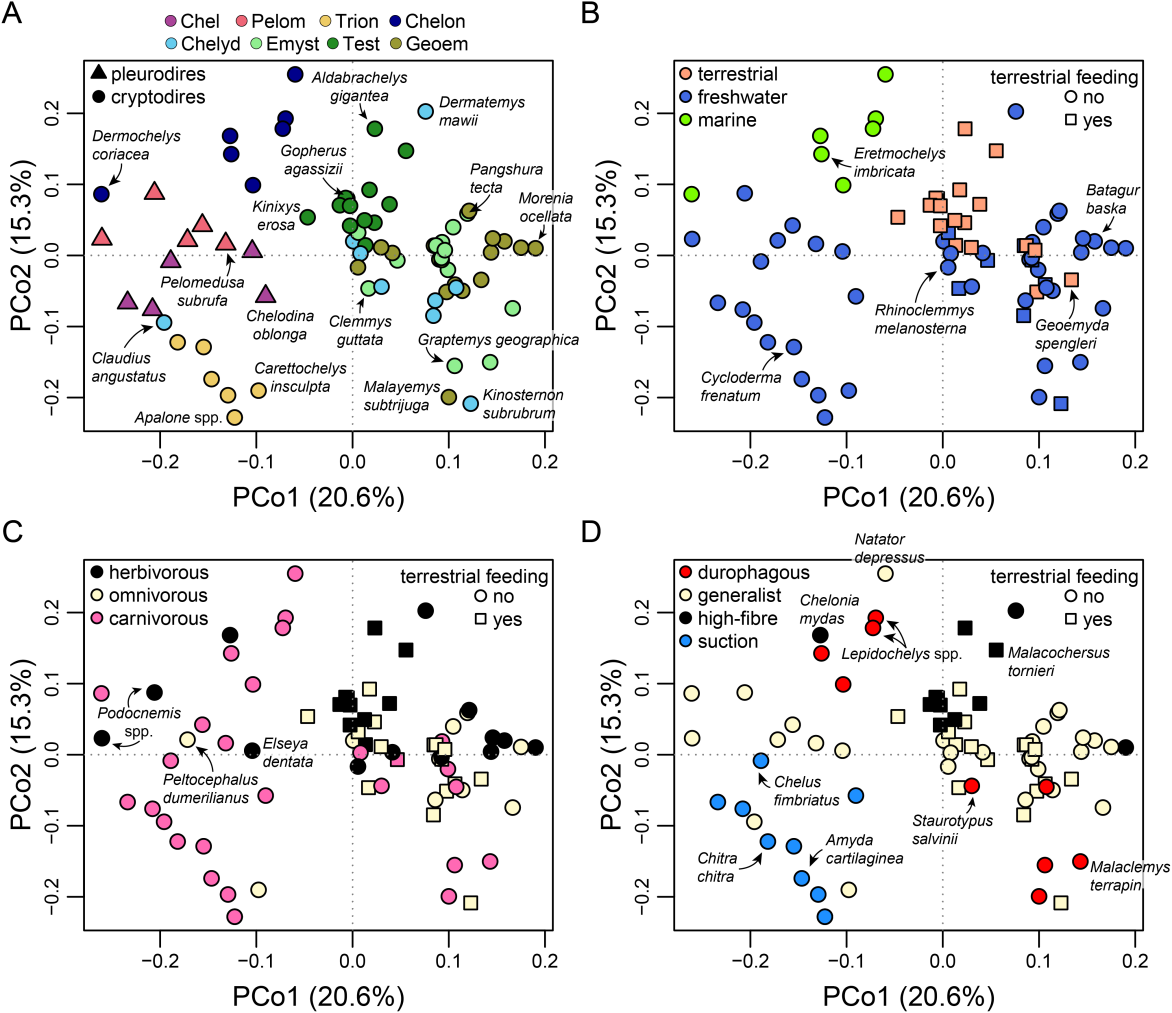
PCoA morphospace of mandibular character scorings based on first two principal coordinate axes, with various grouping superimposed on the data. (A) Major turtle clades. (B) Habitat ecology. (C) Primary diets. (D) Specialized diets. Note the different color and point‐symbol keys per panel. See Figure S13 in File S2 for repeated plots in which all species are indicated by numbers. Clade abbreviations: Chel, Chelidae; Chelon, Chelonioidea; Chelyd, Chelydroidea; Emyst, Emysternia; Geoem, Geoemydidae; Pelom, Pelomedusoides, Test, Testudinidae; Trion, Trionychia.

Ordination of discrete mandibular character data results in a morphospace (Figure 7) in which individual turtle clades can be distinguished a lot better than in the PCA morphospace based on functional measurements (Figure 6). This likely reflects the nature of the data, as the discrete characters used have been shown to have phylogenetic signal and include characters that are synapomorphic at various clade‐levels (Evers et al. 2023). It also shows that most turtle clades have similar mandibular scorings among their species (e.g., chelonioids, pelomedusoids, chelids, trionychians, testudinids), despite differences in functional measurements that lead to clade dispersion in the PCA based on functional measurements for the same species. PCo axes 1 and 2 summarize 35.9% of the total variance, with 20.6% and 15.3% explained by the first and second axes (Figure 7), respectively. It takes four PCo axes to explain 50% and 14 PCo axes to explain 90% of the variance, despite a matrix size of 51 characters. The first 18 axes each explain more than 1.0% of the total variance (File S2: Table S1). PCo axis 1 separates the clades Trionychia, Chelidae, Pelumedusoidea, and Chelonioidea at negative values from testudinoids and chelydroids, which have predominantly positive PCo1 scores (Figure 7A). Chelonioids have slightly higher PCo1 values than the other clades with predominantly negative PCo1 values, but their morphospace is strongly extended into highly negative values by the leatherback sea turtle, *Dermochelys coriacea* (Figure 7A). Although trionychids, chelids, and pelomedusoids have overlapping PCo1 values, these clades are separated along PCo2, with chelonioids having the highest PCo2 values and trionychians the lowest (Figure 7A). Chelids and pelomedusoids are spread across the mid‐range of PCo2 values. Although they fall closely together, pelomedusoids assume higher PCo2 values than chelids. Overall, pleurodires occupy a relatively small area of the morphospace compared to cryptodires (Figure 7A). Among testudinoids, testudinids form a cluster of datapoints at slightly lower PCo1 and slightly higher PCo2 values than emydids and geoemydids, which have completely overlapping ranges (Figure 7A). Chelydroids expand over the entire range of testudinoid values, and expand to strongly negative PCo1 values with a single data point, the kinosternid chelydroid *Claudius angustatus* (Figure 7A). This is by virtue of the pleurodire‐like convex hemispherical jaw joint in this taxon (Evers et al. 2023). Each of the groups that are well separated from other clades exhibits a large number of mandibular synapomorphies (Evers et al. 2023).

The patterns of ecology distributions can largely be explained by taxonomy. All marine turtles in our sample belong to a single clade (i.e., Chelonioidea) and thus have a relatively confined region among the PCoA colored by habitat (Figure 7B). However, whereas *Dermochelys coriacea* is strongly distinct from hard‐shelled cheloniids in the functional PCA data (Figure 6), it is closer to its relatives in the PCoA data (Figure 7A), as it shares numerous mandibular synapomorphies with cheloniids (Evers et al. 2023). Similarly, terrestrial turtles cluster closely together (albeit overlapping with aquatic turtles; Figure 7B), as these are composed of testudinids and various geoemydids and emydids (Figure 7A). Freshwater aquatic turtles extend across the entire morphospace (Figure 7B), as they contain species from nearly all clades (with the exception of chelonioids and testudinids; Figure 7A), therefore covering the full range of realized character state combinations. Terrestrially feeding turtles are confined to relatively high PCo1 values, but they span the full range of PCo2 values (Figure 7B–D). Among primary diets, carnivorous turtles span the largest area of the morphospace (Figure 7C), as carnivory is present in such distinct clades as chelonioids, pleurodires, trionychians, and testudinoids (Figure 7A). The clearest visual separation of ecological clusters is seen among specialized diets, in which high‐fiber herbivores and suction feeders have non‐overlapping ranges (Figure 7D). Suction feeders are fairly isolated at low PCo1 and PCo2 values (Figure 7D). Durophagous turtles show at least two separate clusters (Figure 7D), which can again be explained taxonomically: this feeding behavior primarily occurs among chelonioids, which are fairly distinct as a clade in morphospace, among chelydroids, and various emydids and geoemydids (Figure 7A). In addition, some trionychids are at least capable of durophagy, although we have classified trionychids herein either as suction feeders or as generalists (Hermanson et al. 2022).

### 3.4 Turtle Mandibular Jaw Disparity

We used the same disparity metric, sum of variances, to compare functional disparity with character disparity using statistical tests for various taxonomic and ecological subgroups of turtles (Table 3). The tests were hereby designed to answer evolutionary questions introduced above (see Methods and Table 1).

**TABLE 3:** Results from pairwise comparisons of functional and character disparities based on sum‐of‐variances extracted from morphospace coordinates.

Pairwise two‐sided Wilcoxon comparison	Functional disparities		Character disparities	
	*W*	*p*	*W*	*p*
Clade‐wise comparisons				
Cryptodires versus pleurodires	138,585	< 0.001	658,914	< 0.001
Primary diets				
Carnivorous versus omnivorous	1,000,000	< 0.001	1,000,000	< 0.001
Omnivorous versus herbivorous	0	< 0.001	85,138	< 0.001
Carnivorous versus herbivorous	954,367	< 0.001	995,350	< 0.001
Terrestrial feeding				
Yes versus no	999,979	< 0.001	1,000,000	< 0.001
Specialized diets				
Generalists versus high‐fiber herbivores	255,082	< 0.001	856,096	< 0.001
Generalists versus durophages	309,900	< 0.001	188,423	< 0.001
Generalists versus suction feeders	21,922	< 0.001	682,294	< 0.001

*Note:* *W* is the test‐statistic for the Wilcoxon test. Raw *p*‐values as returned from disparity analyses are available in File S5.

**FIGURE 8:**
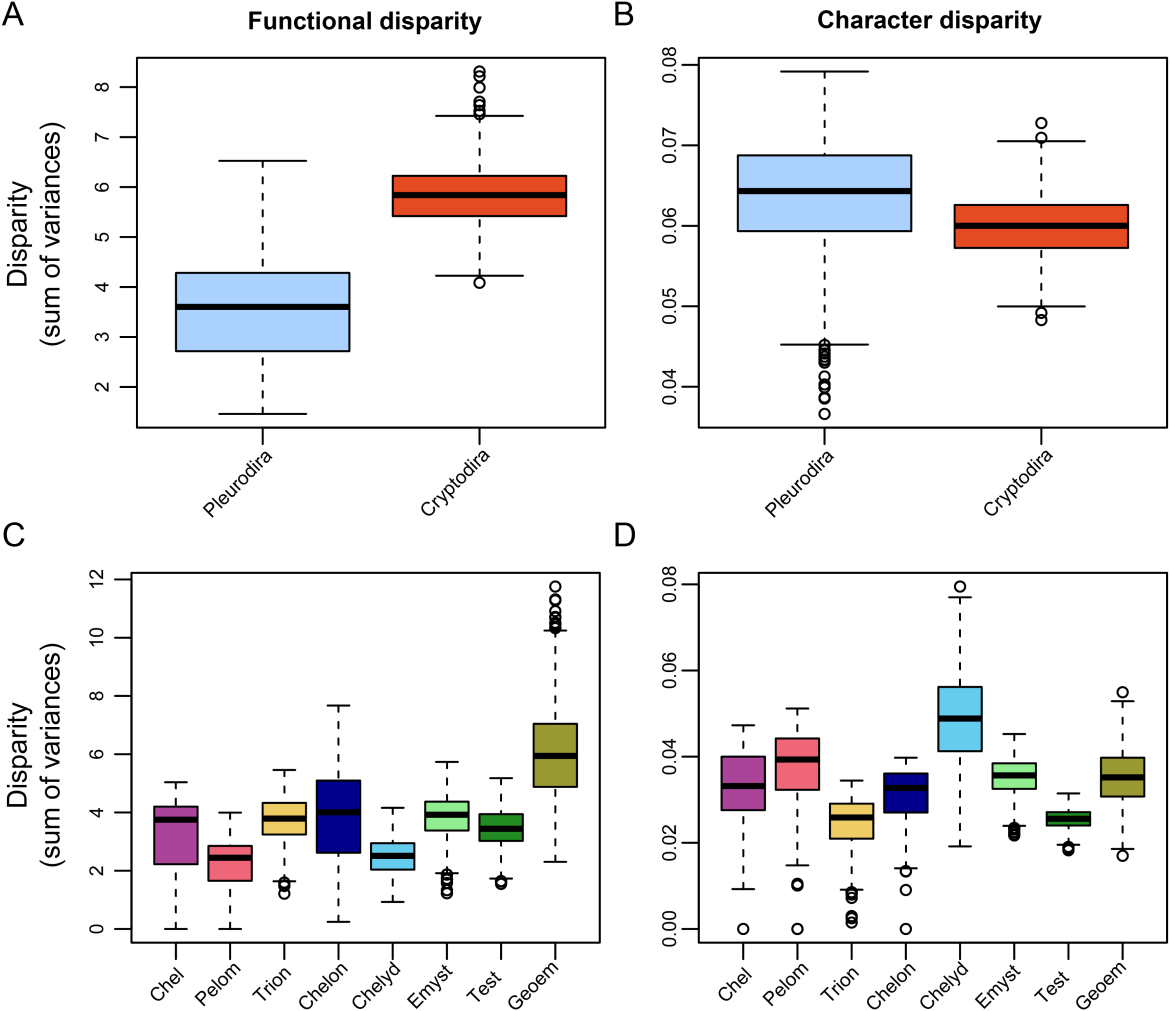
Boxplots comparing functional and character disparities of turtle mandibles for taxonomic groups or clades. Note that A–B reflect the disparity question 1 laid out in Table 1. Black bar within boxplots represents the median values, and boxes themselves indicate the interquartile range. Column A, C shows functional disparity and column B, D shows character disparity. Row A–B shows a cryptodiran‐pleurodiran comparison, row C–D shows clade‐wise disparities. Clade abbreviations: Chel, Chelidae; Chelon, Chelonioidea; Chelyd, Chelydroidea; Emyst, Emysternia; Geoem, Geoemydidae; Pelom, Pelomedusoides, Test, Testudinidae; Trion, Trionychia.

Functional and character disparity patterns differ on various clade‐levels (Figure 8; Table 3). This is particularly evident when comparing the two major extant turtle lineages, i.e., cryptodires and pleurodires (Figure 8A,B): Pleurodires have significantly lower functional disparity than cryptodires (*p* < 0.001; Table 3), but significantly higher character disparity (*p* < 0.001; Table 3). The differences between functional and character disparity are also apparent at less inclusive clade‐levels (Figure 8C,D). Whereas geoemydids and chelonioids have the highest and pelomedusoids and chelydroids the lowest functional disparity among turtles (Figure 8C), chelydroids and chelonioids have the highest and trionychians and testudinids the lowest character disparity (Figure 8D). Among pairwise clade comparisons, nearly all clades show significant differences to one another (exception for functional disparity: chelids and testudinids; exception for character disparity: emysternians and geoemydids, as well as trionychians and testudinids; File S5). While some clades have narrow ranges of disparity (e.g., trionychians, testudinids) in both data types (Figure 8C,D), others show wide ranges in both (e.g., chelonioids; Figure 8C,D) or differ between data types (e.g., chelydroids; Figure 8C,D). The high character disparity in chelydroids (Figure 8D) is caused by the inclusion of the unusual mandible of *Claudius angustatus* , as well as *Dermatemys mawii* , which also has a high number of distinct discrete features (Evers et al. 2023). Similarly, high disparity values among chelonioids are partially due to *Dermochelys coriacea* .

**FIGURE 9:**
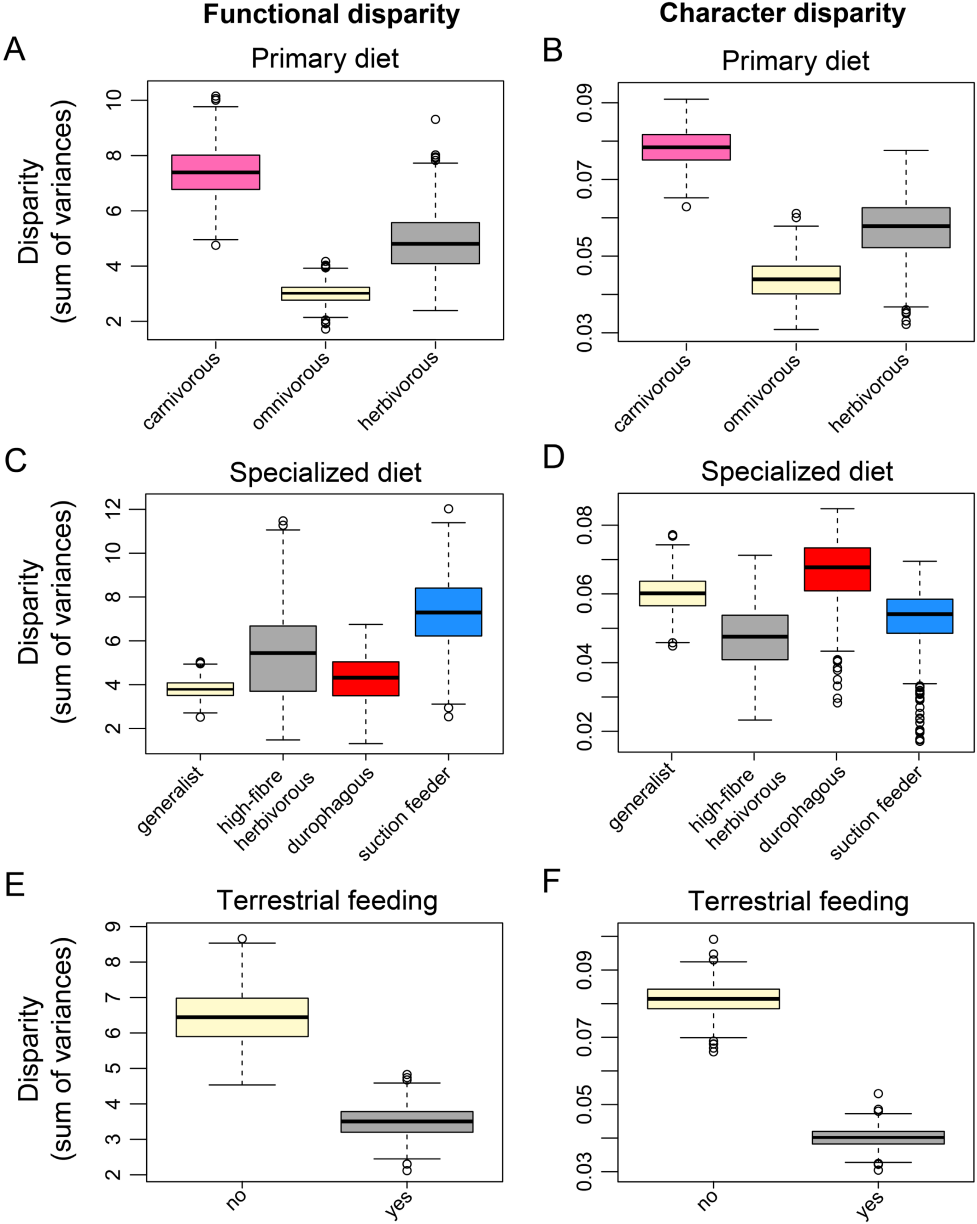
Boxplots comparing functional and character disparities of turtle mandibles for ecological groups. Note that these reflect the disparity questions laid out in Table 1. Black bar within boxplots represents the median values, and boxes themselves indicate the interquartile range. Column A, C, E shows functional disparity and column B, D, F shows character disparity. Row A, B shows primary diet, row C, D shows specialized diets, row E, F shows terrestrial feeding capacity.

Disparity tests for ecological subgroups of turtles show that turtles with different primary diets occupy significantly different volumes in morphospace (Figure 9A,B). Hereby, functional and character disparity patterns agree in showing that carnivorous turtles have significantly higher disparity than herbivorous turtles (*p* < 0.001; Table 3), which in turn have significantly higher disparity than omnivorous turtles (*p* < 0.001; Table 3). Turtles with the capacity to feed on land have significantly lower functional and character disparity than turtles that do not (*p* < 0.001; Table 3; Figure 9C,D). Dietary specialists (i.e., high‐fiber herbivores, durophages, suction‐feeders) all have significantly higher functional disparity than generalists (*p* < 0.001; Table 3; Figure 9E). Among those specialists, suction feeders have the highest functional disparity of all (Figure 9E). However, these functional disparity patterns are not mirrored in character disparity (Figure 9F). Here, only durophagous turtles have significantly higher character disparity than generalists, whereas high‐fiber herbivores and suction feeders both have significantly lower character disparity than generalists (*p* < 0.001; Table 3; Figure 9F).

## 4 Discussion

### 4.1 Interpretation of Functional Measurements

Functional measurements like the ones employed in our study have frequently been used to characterize the functional disparity of various gnathostome jaws (e.g., Maynard‐Smith and Savage 1959; Emerson 1985; Westneat 2003; Kammerer, Grande, and Westneat 2006; Stayton 2006; Rayfield et al. 2007; Anderson 2009a; Sakamoto 2010; Anderson et al. 2011; Anderson, Friedman, and Ruta 2013; Stubbs et al. 2013; Button, Rayfield, and Barrett 2014; Mallon and Anderson 2015; Nabavizadeh 2016; MacLaren et al. 2017; Navalón et al. 2019; Benevento, Benson, and Friedman 2019; Ma et al. 2020, 2022; Morales‐García et al. 2021; Singh et al. 2021, 2024; Meade and Ma 2022; Johnson et al. 2022; Schade et al. 2022; Foffa, Young, and Brusatte 2024). These measurements, especially mechanical advantages related to jaw‐closing (i.e., AMA and PMA) and jaw opening (i.e., OMA), are based on lever mechanics and provide biomechanical expectations grounded on physics. For example, high closing mechanical advantages signify a good force transmission of the input force exerted onto the food item, and are thus correlated with bite force (Westneat 1994, 2003; Stayton 2006). However, high closing mechanical advantages also indicate a slower bite, such that AMA and PMA are commonly interpreted as a trade‐off between bite force and bite speed (Westneat 1994, 2003; Stayton 2006). Similarly, jaw OMA correlates with force transmission of the jaw depressor (=extensor) muscles, whereby high values come at the expense of opening speed (Westneat 1994, 2003). Thus, expected biomechanical performances of jaws in terms of force and speed are governed by different jaw proportions that alter the ratios between in‐ and out‐levers. Due to the inverse relationships of force transmission and speed, the mechanical advantage measurements are more difficult to interpret than our other functional variables, which are easier to relate with specific feeding modes (e.g., reduction of triturating surfaces with reduction of importance of food processing).

We used general observations of turtle diets and feeding strategies as a guide to formulate hypotheses about the biomechanical properties we expect in various turtle ecologies. The force transmission vs. speed trade‐off described above hereby generally led to expectations that hunters (i.e., specifically suction feeders) have weak but fast jaw opening and closing dynamics, whereas turtles eating tough but slow foods (i.e., durophagous or herbivorous turtles) show strong but slow jaw movements. However, the results of our mechanical advantage measurements do not always fit these expectations. Specifically, high mechanical advantage values in chelids as primarily suction‐feeding turtles indicate unexpectedly high force transmission at the expense of speed. Similarly, trionychids have high OMAs, again indicating good force transmission but low speed despite their active hunting style that encompasses suction. Some of the other biomechanical measurements also give unexpected results, for instance, durophagous turtles having relatively low SMOI values suggesting low stress resistance and relatively elongate jaws. Nevertheless, some of these initially surprising measurement results can possibly be explained. For example, it makes sense that durophagous turtles and suctions feeding turtles fall at the opposite ends of the spectrum of second moment of inertia values. The low values of durophages indicate that it is not only the triturating surface that is broadened in these turtles, but also the main beam of the mandibular ramus that buttresses the triturating surface. Durophages have dentary cross‐sections that are mediolaterally broad but dorsoventrally low, resulting in low SMOI values. While it is counterintuitive that durophagous turtles should have particularly low resistance to dorsoventrally exerted stress, these turtles accommodate the food item over a much broader surface, thus possibly distributing stress mediolaterally. The jaw morphology of durophagous turtles is relatively rare among tetrapods (e.g., see moradisaurine captorhinids: Modesto et al. 2018), which usually have dentary rami that are dorsoventrally taller than they are mediolaterally wide. The initial use of the second moment of inertia measurement by Anderson (2009a) was also used on taxa with the more common jaw morphology of dorsoventrally tall dentaries. Although it is in principle possible that the biomechanical interpretation of this measurement cannot easily be transferred to taxa that encompass both taller‐than‐wide and wider‐than‐tall morphologies, there are also potential biomechanical factors that affect jaw stiffness that are not recorded in our approach. For instance, Figure 1 shows density differences in the compactness (i.e., proportion of air‐filled spaces within the bone cross‐section with regard to dense bone) between *Eretmochelys imbricata* as a taxon with high SMOI values and low bone compactness (Figure 1D) compared to *Graptemys geographica* with low SMOI values and a dense cross‐section (Figure 1F). These differences in the material properties are a major factor not examined in our study. Suction feeders, as well as herbivorous turtles, have mandibles that are generally narrow but dorsoventrally tall. High SMOI values indicate high mechanical resistance and thus high resistance to deformation stress in the dorsoventral axis (Anderson 2009a). Herbivores, but also suction feeding turtles may thus have high stress resistance because food items exert high stress on a small surface area of the mandible, for example the labial ridges during the shearing of fibrous food items. Contrary to the examples above, many functional measures also conform to our expectations. For example, our expectations for herbivores and especially high‐fiber herbivores are often met, as these turtles have high PMA values with better force transmission at lower jaw‐closing speeds, high resistance to vertically enforced stress, and relatively short jaws. Relative triturating surface width also behaves as expected, with suction feeding turtles showing reduced surfaces (e.g., Lemell et al. 2010; Joyce, Rollot, et al. 2021) and durophagous turtles having wider surfaces (e.g., Ferreira et al. 2015).

**FIGURE 10:**
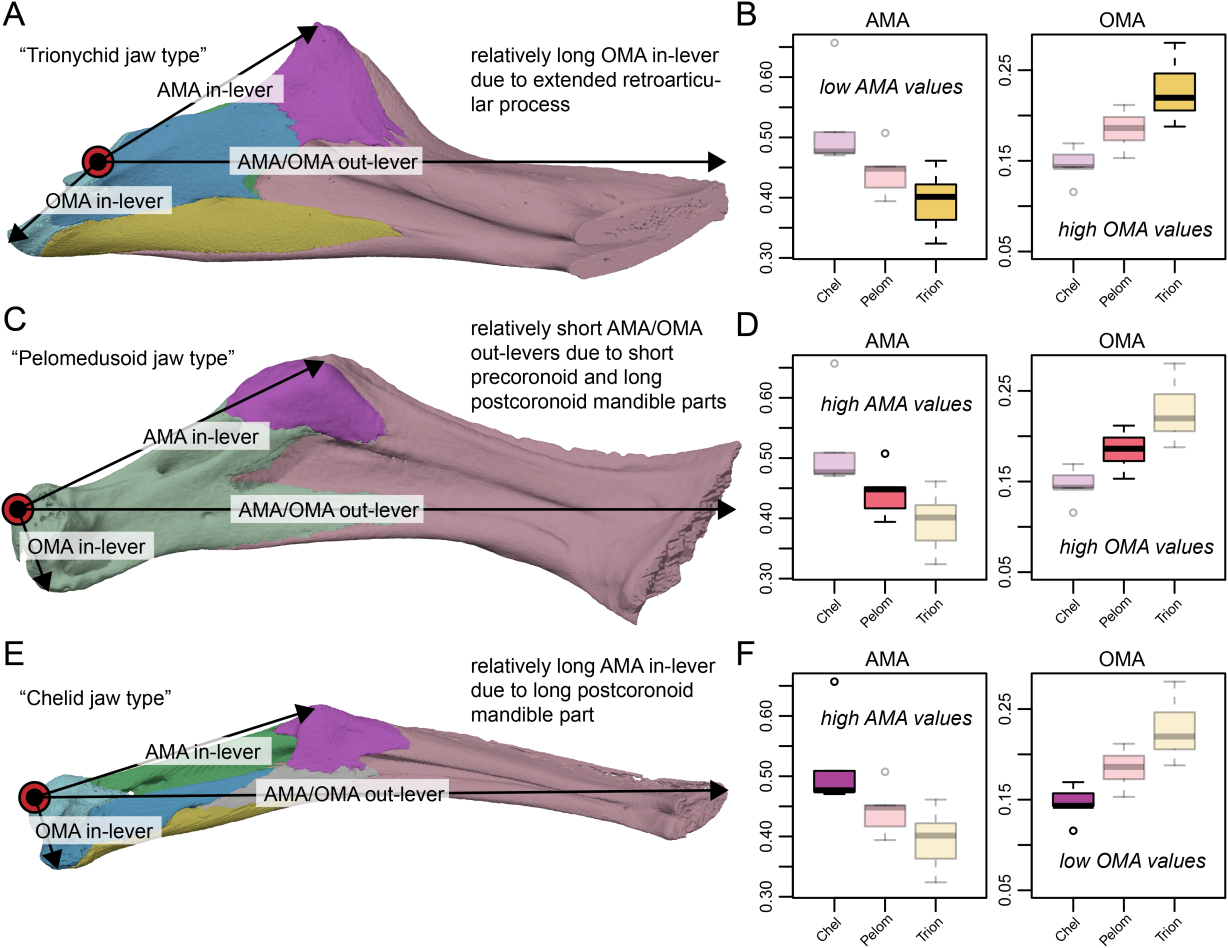
Explanation of turtle “jaw types” that result from extreme proportional differences of distances relevant for lever mechanics. (A) In‐ and out‐levers for anterior and closing mechanical advantage for the “trionychid jaw type”, illustrated by *Amyda cartilaginea* (FMNH 244117). (B) Resulting mechanical advantages. (C) In‐ and out‐levers for anterior and closing mechanical advantage for the “pelomedusoid jaw type”, illustrated by *Pelomedusa subrufa* (FMNH 17161). (D) Resulting mechanical advantages. (E) In‐ and out‐levers for anterior and closing mechanical advantage for the “pelomedusoid jaw type”, illustrated by *Hydromedusa tectifera* (SMF 70500). (F) Resulting mechanical advantages. Note that A, C, E are mandibles in medial view scaled to approximately the same sizes with coronoid processes and mandibular tips aligned at the same vertical position, highlighting proportional differences. Black bar within boxplots represents the median values, and boxes themselves indicate the interquartile range. Clade abbreviations: Chel, Chelidae; Pelom, Pelomedusoides; Trion, Trionychia.

Our results show various combinations of high and low closing mechanical advantages with high and low OMAs. In an attempt to explain the distribution of these values measured for turtles, we explored the various jaw proportions that are relevant for the in‐ and out‐levers. Hereby, we find that extreme variations of any one lever measurement can explain jaw shapes that result in interesting combinations of biomechanical measurements, which we describe as different “jaw types” below that are named for the clade that principally displays this type, shown in Figure 10. (1) In the “trionychid jaw type”, the retroarticular process is extremely elongated (increasing the OMA in‐lever), which results in proportionally high OMA values at relatively low AMA values that can be seen among trionychians (Figure 2A,C). This combination implies relatively slow opening jaw speeds but relatively weak yet fast bites. (2) In the “pelomedusoid jaw type”, the pre‐coronoid part of the mandible is relatively short, resulting in short AMA/OMA out‐levers. This causes elevated AMA and OMA values at the same time, as realized among pelomedusoids (Figure 2A,C). This indicates powerful but slow jaw opening and closing. (3) In the “chelid jaw type”, the postcoronoid part of the jaw is elongated without hypertrophied retroarticular process (increasing the AMA in‐lever), resulting in AMA values that are disproportionally high relative to the OMA values, as seen in chelids (Figure 2A,C). For chelids, this implies average jaw opening speeds but low closing speeds at high force transmission.

Not all turtles can be fit into one of these models because not all turtles have disproportionally lengthened or shortened one of the relevant jaw sections. Some turtles are also hard to fit into the general framework of force and speed trade‐off. Snapping turtles (chelydrids), for instance, are known to be powerful yet fast biters (Herrel, O'Reilly, and Richmond 2002; LaGrange et al. 2023), whereby biting speed can be adjusted to prey type (Lauder and Prendergast 1992). According to our measurements, these turtles have average jaw opening values but high AMA and PMA values. This means that their biting force transmission is high and decreases little toward the anterior end of the jaw, but also implies slow bites. These observations possibly suggest that the jaws of chelydrids are primarily selected for high force, whereby expenses in speed may be compensated for in different ways, such as elongation of mandibular hooks that effectively reduce the gape angle with which food items are caught, causing them to be quickly trapped (LaGrange et al. 2023), muscle physiology, or fast neck movements (Anderson 2009b).

Chelids and trionychids are the primarily suction‐feeding clades of turtles, but they have fundamentally different biomechanical jaw arrangements according to our data. This indicates that these clades have different biomechanical feeding modes that both allow an active, suction‐feeding hunting style. The low opening and closing speeds implied by our measurements for chelids are unexpected, as chelid feeding observations show that ingestion of food items happens fast (e.g., Lemell et al. 2002) and as adductor jaws of at least *Chelus fimbriatus* have been described to produce high closing velocities despite being poorly developed (Lemell et al. 2002, 2010). However, high OMA values do not necessarily contradict high opening speeds because opening jaws rapidly under water also requires a considerable amount of force. Opening speeds required for suction feeding can be achieved despite a mechanical jaw lever design optimized for forceful action, for example by muscle physiology. Our biomechanical parameters are modeled after simple lever mechanics, which certainly oversimplify the feeding process in turtles and completely ignore the action of muscles. The length, arrangement, and type of muscle fibers all influence the contraction speed of muscles (e.g., Schumacher 1973; Ferreira and Werneburg 2019). Thus, although lever mechanics imply slow speeds for chelids, this may be compensated for by the specific muscle anatomy of the animals, which we did not investigate. In addition, hyolingual and also neck movements may play a larger role in facilitating the speed of the feeding event than the jaws do, particularly as the forward strike of the neck plays an active role in opening the jaws in *Chelus fimbriatus* (Lemell et al. 2002) and as the forward strikes of chelids generally have high velocities (Aerts, van Damme, and Herrel 2001). It is well documented that the hyolingual movements facilitate most of the suction force (Lemell et al. 2002, 2010, 2019). Thus, the high AMA and relatively high OMA values possibly suggest that effective force transmission is important in the feeding cycle, too, potentially to achieve the high angles of mouth opening recorded at 80° for *Chelus fimbriatus* (Lemell et al. 2002). We hypothesize that high closing force transmission of chelids plays a role in keeping the jaws shut after the prey was fully ingested into the mouth cavity. This may be important because the high SMOI values for chelid jaws suggest that while they can withstand high vertical forces, mediolateral forces exerted by struggling prey items may be problematic. The slow opening speed indicated for trionychids is also unexpected for suction feeders. Possibly, the high force transmission in opening the jaw caused by the elongated muscle attachment sites for jaw opening muscles plays a supporting role in opening the disproportionally long jaws of trionychids while submerged. Also, trionychids have protracted necks and make contact with their prey before initiating the gape‐cycle, such that a bow wave that requires compensatory suction force is avoided (Anderson 2009b). Possibly, this allows for relatively lower jaw opening speeds. Our results reinforce the idea that suction feeding among turtles is achieved using very different skull proportions (e.g., Hermanson et al. 2022), hyoid morphologies (Van Damme and Aerts 1997; Jorgewich‐Cohen et al. 2024), as well as neck movements and proportions (e.g., Van Damme and Aerts 1997; Aerts, van Damme, and Herrel 2001; Lemell et al. 2002, 2010; Herrel et al. 2007; Van Damme and Aerts 1997; Joyce, Rollot, et al. 2021).

The various combinations of OMA and AMA values we observed for turtles reinforce the notion that opening and closing mechanical advantages are not correlated (e.g., Anderson 2009a): Despite sharing the same out‐lever (i.e., distance from fulcrum to anterior end of mandible), AMA and OMA have in‐levers that vary independently from one another. The expectation that AMA and PMA are correlated (Anderson 2009a) is matched in our turtle data. However, this correlation of AMA and PMA for turtles (*R* = 0.44; *p* = 6.7 × 10^−5^) is lower than reported for some other groups (e.g., arthrodires: *R* = 0.95; Anderson 2009a; Mesozoic pseudosuchians: *R* = 0.7: File S2: Figure S8 using data from Stubbs et al. 2013), indicating that turtles have a relatively high discrepancy between bite force and bite speed between anterior and posterior bites. This indicates that turtles have great disparity in their pre‐coronoid jaw length, as this length is determining differences in the AMA and PMA out‐levers. The ranges of PMA values indicate that turtles achieve very good (i.e., near one‐to‐one) force transmission at the posterior end of the jaw, whereas the force transmission is a lot lower at anterior biting points, with strong differences between groups (see chelydrid example above). This is primarily caused by pleurodires, in which both chelids and pelomedusoids have proportionally larger AMA values than cryptodires, with the exception of chelydroids (Figure 2A,B). Anatomically, this can be explained by the long postcoronoid‐jaw length and relatively short dentary ramus of pleurodires and chelydroids that cause relatively long in‐levers (for both PMA and AMA) but a relatively short AMA out‐lever.

Turtles have statistically significantly higher AMA and PMA values than pseudosuchians (Stubbs et al. 2013; File S2: Figure S9) based on *t*‐test comparisons (mean_turtle‐AMA_ = 0.42; mean_pseudosuchia‐PMA_ = 0.19; *T*_AMA_ = 23.1; *p*_AMA_ < 0.001; mean_turtle‐PMA_ = 0.96; mean_pseudosuchia‐PMA_ = 0.38; *T*_PMA_ = 32.7; *p*_PMA_ < 0.001). This is the result of comparatively short pre‐orbital regions in turtles compared to pseudosuchians with elongated rostra, which affect the proportion of jaw‐closing out‐levers. Increased force transmission seen in turtles can possibly be important because of their comparatively small body size. Smaller body sizes also imply smaller head sizes, and bite force strongly scales with head size (Herrel, O'Reilly, and Richmond 2002; Erickson, Lappin, and Vliet 2003; Herrel et al. 2006; Pfaller, Gignac, and Erickson 2010; Marshall et al. 2012; LaGrange et al. 2023). Thus, in order to retain high bite‐forces at small head (as a consequence of small body) sizes that are beneficial to their dietary adaptations, turtles require extremely high jaw‐closing mechanical advantages. The relatively short jaw‐closing out‐levers, which also correspond to comparatively short pre‐orbital regions in the cranium of turtles compared to other amniotes (see, e.g., Ferreira and Werneburg 2019), may thus be an adaptation to mitigate smaller bite‐forces at smaller body sizes. Pseudosuchians, on the other hand, can make due with lower jaw‐closing mechanical advantages because high bite‐forces can be achieved nevertheless due to their large body size and muscle arrangements. The hypothesis that turtles have jaws optimized to retain high bite‐forces at small body sizes is supported by the relationships of AMA with head size among turtles: There is a significant, albeit weak negative correlation showing that smaller turtles tend to have higher force transmissions for anterior mechanical advantage (*R* = −0.33; *p* = 0.003; File S2: Figure S7).

### 4.2 Decoupling of Functional and Character Disparity Patterns in Turtle Mandibles

Functional variation of turtle jaws is evolutionarily decoupled from discrete character disparity. This is apparent both in taxonomic as well as ecological groups. High functional but low character disparity occurs in cryptodires and the opposite in pleurodires. As sister clades have the same evolutionary time to accumulate variance, these differences imply either heterogeneous evolutionary rates in these groups (e.g., Brusatte et al. 2008, 2012; Lloyd, Wang, and Brusatte 2012; Wang and Lloyd 2016) or selective extinction of morphotypes in one group but not the other (Friedman 2009; Grossnickle and Newham 2016; Pimiento et al. 2020; Puttick, Guillerme, and Wills 2020; Cole and Hopkins 2021). There is plausible evidence for both effects among turtles. Cryptodires today explore a broader spectrum of ecological niches (Pritchard 1979; Ernst and Barbour 1989) and have a wider geographic range (TTWG 2021). For example, several independent lineages of cryptodires have durophagous diets (e.g., chelonioids, geoemydids, emydids, kinosternids). Convergent functional adaptations to durophagy among these clades result in relatively low functional disparity, while this ecological group has a high character disparity reflecting the distinct phylogenetic lineages that evolve durophagy. This discrepancy can be explained when several character combinations can convergently lead to similarities in triturating surface design. Cryptodires also have larger phylogenetic diversity than pleurodires (e.g., Thomson, Spinks, and Shaffer 2021; Joyce, Anquetin, et al. 2021). Thus, their increased functional disparity may reflect adaptive processes associated with higher evolutionary rates, but may also be an effect of phylogenetic diversification (e.g., Melstrom et al. 2021). Pleurodire functional disparity may additionally be impacted by extinction of past morphotypes, as has been documented for various gnathostomes (e.g., Wilberg 2017). For turtles, Hermanson et al. (2022) documented that bothremydids, a highly diverse and disparate group of extinct pleurodires, explored cranial shapes not found among extant turtles. The possible effect that the inclusion of fossil mandibles may have on disparity is expected to be higher for functional disparity than for character disparity. This is because bothremydids only expand the disparity range of cranial shape, but not cranial topologies of extant pleurodires (Miller et al. 2023). Topology tracks the sutural connections of skull bones, and thus has a similar structure to discrete morphological characters, which usually encode the same type of information (among additional, non‐topological variation).

Cryptodires achieve a higher range of functional disparity at a lower exhaustion of character state‐combinations. Among ecological groups, this can also be seen in suction feeders. Conversely, pleurodires as well as some ecological groups (e.g., generalists, durophages) show high variation in terms of character states, but this does not result in greater functional variation. The high character disparity of generalists can be explained by the phylogenetic breadth at which this dietary category is observed, which is similar in durophages as already argued above. As the discrete mandibular character data show strong phylogenetic signal (Evers et al. 2023; this study), cryptodires may be constrained to a limited combination of character states and achieve high functional disparity instead by strong variations of proportions. Similar discrepancies between shape and topology have been observed for the turtle skull ontogeny, where large shape differences are accumulated during ontogeny despite low variance in topological connections of bones (Miller et al. 2023). For ecological groups, fundamentally different shapes can also result in adaptations for the same diets. Specifically, testudine suction feeding is observed among turtles with broad and short skulls (e.g., chelids) but also among long and narrow skulls (e.g., trionychids). While the biomechanics of these differently shaped mandibles are different and thus results in a high functional disparity, many discrete traits (e.g., narrow triturating surfaces, reduction of triturating ridges, etc.) are convergently present in this ecological group.

The decoupling of functional and character disparity is also evident in some ecologies. Hereby, the low functional disparity of generalists is surprising, because generalists could be expected to experience less selection pressure to maintain a specific jaw shape that is adapted to a specialization. Although this seems to be true for the character disparity, which is likely high due to the phylogenetic breath of generalists, our results seem to indicate that it is indeed the ecological specialization that facilitates departure from a mean generalist shape in terms of biomechanical proportions of the mandible. It is possible that the generalist diet requires the evolutionary maintenance of an “average mandible”, as biomechanical specializations in the mandible are incompatible with a broad dietary spectrum.

Despite widespread decoupling of functional and character disparity among our taxonomic and ecological comparisons, sometimes functional and character disparities also agree. For example, terrestrially feeding turtles have lower functional and character disparity than aquatic turtles. This likely simply reflects the wider range of specializations seen in aquatic turtles, as all terrestrially feeding turtles are herbivores or omnivores and as the most extreme specializations (durophagy, suction feeding) are seen among carnivores (or omnivores).

The decoupling of functional and character disparity observed here, as well as further differences compared to shape disparity measured by landmarks (e.g., Hermanson et al. 2022) or topological disparity (Miller et al. 2023), highlight that disparity trends are dependent on the way we measure them. Thus, the choice of data underlying the disparity computation should be selected with care and tailored to the evolutionary question at hand, just as the disparity metric used (Guillerme et al. 2020).

### 4.3 Decoupling of Mandibular and Cranial Disparity Patterns

Our data also suggest that the mandible has disparity patterns that are decoupled from cranial disparity patterns. Specifically, the higher functional jaw disparity of cryptodires compared to pleurodires contrasts with cranial disparity patterns (Foth, Ascurranz, and Joyce 2017). Mandibular‐cranial decoupling is also apparent from individual taxa with unusual biomechanical proportions or character state combinations. These species cause high mandibular disparities among turtle subclades. Chelonioid functional and character disparity and chelydroid character disparity are significantly increased by species with unusual morphologies, specifically *Dermochelys coriacea* , *Claudius angustatus*, and *Dermatemys mawii* . These species expand the morphospace of their parent clades, particularly in the character‐based PCoA morphospace (Figure 7). Nevertheless, unusual mandibular morphologies do not correlate with unusual shapes in the cranium, as none of these species are found to be outliers with regard to their clade's mean shape (Foth, Rabi, and Joyce 2017; Hermanson et al. 2022). In the future, this should further be scrutinized by analyzing mandibular shape quantified using landmarks, as these were the data underlying disparity analyses in Foth, Rabi, and Joyce (2017) and Hermanson et al. (2022), thus allowing for a more direct comparison of mandibular and cranial disparity. Unusual morphologies that we detect using biomechanical measurements and discrete character data may have different evolutionary causes. The skeletal morphology of *Dermochelys coriacea* is distinctly paedomorphic (Nick 1912; Rhodin 1985; Chatterji et al. 2022), and the absence of ossification of various mandibular elements (e.g., coronoid, articular) in adult leatherback sea turtles also suggests paedomorphism in mandibular evolution, as especially the articular is only becoming ossified late during ontogeny in turtles (Evers et al. 2023). Thus, the unusual mandibular morphology of *Dermochelys coriacea* may have primary heterochronic causes. The mandible of *Claudius angustatus* shows high levels of character convergence to the jaws of pelomedusoids (Evers et al. 2023), and *Dermatemys mawii* is distinct from other chelydrids by its strict herbivorous diet and associated morphological innovations, such as labial ridge serrations and the presence of accessory triturating ridges (Evers et al. 2023). Thus, convergence and ecological specializations may increase disparity, as well as developmental constraints. Decoupling of mandibular and cranial evolution has also been documented in several mammalian lineages (Van Cakenberghe, Herrel, and Aguirre 2002; Figueirido et al. 2011; Law et al. 2022).

Our character disparity patterns for various clades confirm known limitations of morphological phylogenetics. We identify trionychids and testudinids as having particularly low character disparity. This supports the notion that many trionychids are very similar in character state composition, implying potentially slow rates of morphological evolution (Evers, Chapelle, and Joyce 2023) and posing problems to phylogenetic inference based on morphology (e.g., Li, Joyce, and Liu 2015; Brinkman, Rabi, and Zhao 2017; Evers, Chapelle, and Joyce 2023; Ponstein et al. 2024). For testudinids, there are also difficulties in placing known fossil material into the framework of extant taxa due to a limited amount of documented morphological variation (e.g., Crumly 1994; Meylan and Sterrer 2000; Vlachos and Rabi 2018; Evers and Al Iawati 2024). Nevertheless, both trionychids and testudinids have overlapping functional disparities with other cryptodires, and skull shape analyses also suggest that shape disparity in trionychids and testudinids is not lower than in other turtle subclades (Hermanson et al. 2022).

## 5 Conclusions

Here, we measure and compare mandibular functional and character disparity across taxonomic and ecological groups of extant turtles. Functional measurements are primarily varied by relative proportional changes in the position of the attachment areas for jaw muscles and triturating surface length, which are important lever arms for jaw opening and closing. Discrete characters have a high phylogenetic signal.

We observe extreme variations in specific jaw levers, summarized in three different “jaw types” (i.e., trionychid, pelomedusoid, chelid), that cause various combinations of closing and opening mechanical advantages. The ranges of posterior mechanical advantage values indicate that turtles achieve very good (i.e., near one‐to‐one) force transmission at the posterior end of the jaw. Although the force transmission is a lot lower at anterior biting points and varies among turtle groups, turtles retain high levels of force transmission at the anterior jaw end compared with other groups (e.g., pseudosuchians). This can possibly be explained as an evolutionary adaptation to achieve high bite forces at small absolute head sizes.

Cryptodires and pleurodires have significantly different functional and character disparities, whereby cryptodires achieve a higher range of functional disparity at a lower exhaustion of character state combinations. This can probably be explained by several reasons, which include the broader ecological and phylogenetic diversity of cryptodires, but also differential extinction patterns leading to the loss of morphotypes in pleurodires specifically. Primary dietary categories of turtles have significantly different disparities, whereby suction feeders show the highest functional disparity caused by two principal skull shapes that are compatible with this feeding mode (long but narrow skulls in trionychids and some chelids; and short but broad skulls in some chelids). Durophages have high character disparity but moderate functional disparity because durophagy causes adaptive similarities in mandible shape that are achieved across a broad phylogenetic spectrum of turtles with different character repertoires. Terrestrially feeding turtles have lower functional and character disparity than aquatic turtles, reflecting the wider range of specializations seen in aquatic turtles. Dietary specialists show larger functional disparity than generalists, but the phylogenetically widespread generalist ecology leads to high character disparity signals in the ecotype. These results show that functional and character disparities are decoupled in turtle mandibles, and comparisons with cranial disparity based on landmarks also suggest differences in mandibular and cranial disparity patterns.

## Author Contributions

**Jasper Ponstein:** conceptualization (equal), formal analysis (equal), investigation (equal), validation (equal), writing – original draft (equal), writing – review and editing (equal). **Guilherme Hermanson:** formal analysis (equal), investigation (equal), methodology (equal), validation (equal), writing – review and editing (equal). **Merlin W. Jansen:** validation (equal), writing – review and editing (equal). **Johan Renaudie:** software (equal), validation (equal), writing – review and editing (equal). **Jörg Fröbisch:** conceptualization (equal), supervision (equal), validation (equal), writing – review and editing (equal). **Serjoscha W. Evers:** conceptualization (equal), formal analysis (equal), investigation (equal), methodology (equal), supervision (equal), validation (equal), writing – review and editing (equal).

## Conflicts of Interest

The authors declare no conflicts of interest.

## Supporting information


Appendix S1.

## Data Availability

We downloaded the 3D models for the turtle jaws from MorphoSource. All R code is provided in Zenodo (10.5281/zenodo.13938962) via GitHub (https://github.com/G‐Hermanson/Turtle‐jaw‐disparity).
