# Supplementary material for: Functional and Character Disparity Are Decoupled in Turtle Mandibles
Source: Ecol Evol. 2024 Nov 13;14(11):e70557. doi: 10.1002/ece3.70557 (PMC11560343; doi:10.1002/ece3.70557)
Supplement: Supplementary file 1 — Appendix S1. [file ECE3-14-e70557-s001.zip › ece370557-sup-0002-FileS2.docx]

**Supplementary information to the article *“Functional and character disparity are decoupled in turtle mandibles”***

Jasper Ponstein^1,2,3^, Guilherme Hermanson^4^, Merlin W. Jansen ^1,2^, Johan Renaudie^2^, Jörg Fröbisch^1,2^ and Serjoscha W. Evers^4^

^1^Humboldt Universität zu Berlin, Unter den Linden 6, 10117 Berlin, Germany

^2^Museum für Naturkunde Berlin, Invalidenstraße 43, 10115 Berlin, Germany

^3^Oertijdmuseum, Bosscheweg 80, 5283 WB Boxtel, Netherlands

^4^Department of Geosciences, University of Fribourg, Chemin du Musée 6, 1700 Fribourg, Switzerland

**
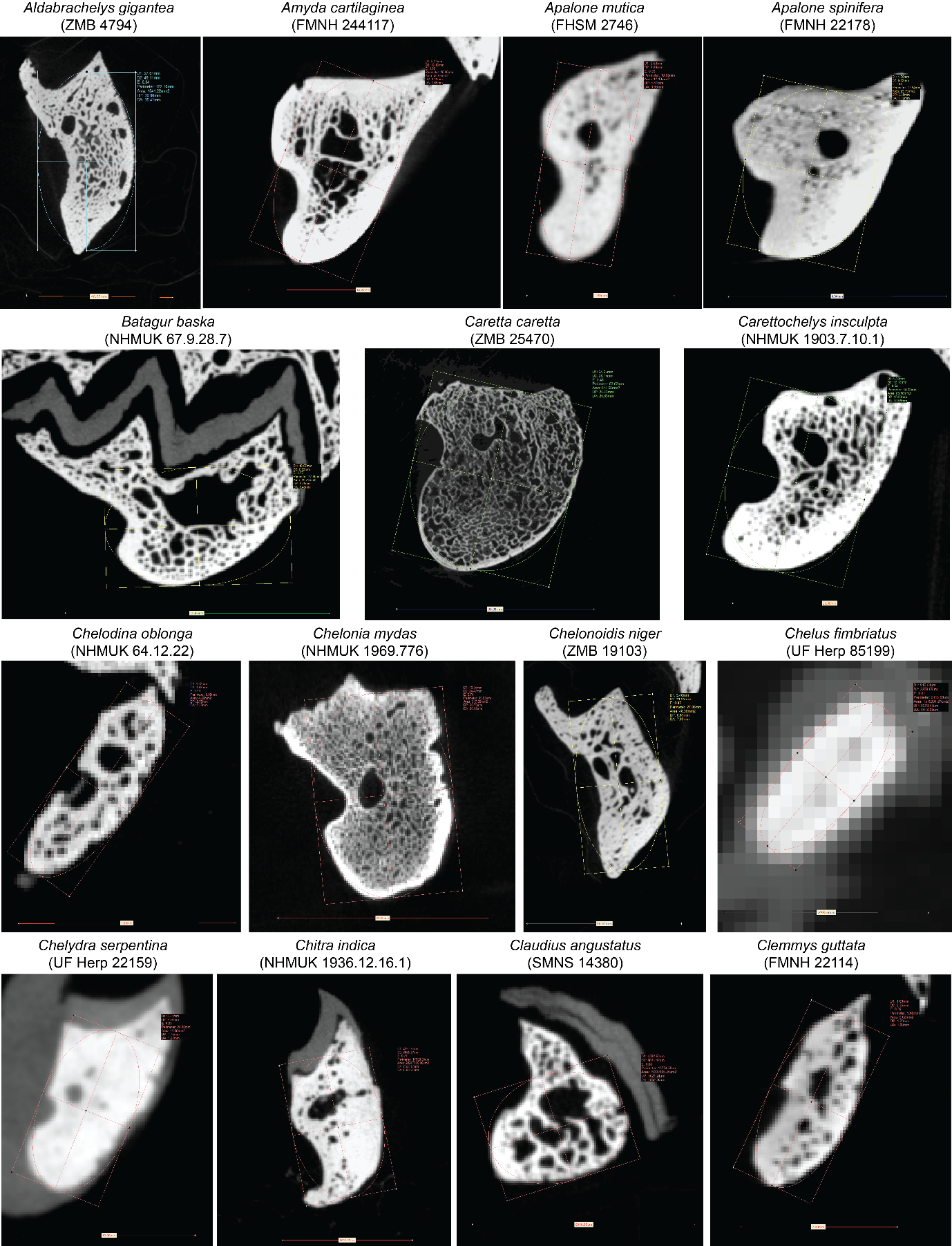
**

**Supplementary Figure S1. Turtle jaw cross-sections.** Cross-sections at the mandibular ramus (see Main Text) used to fit the ellipses to extract Second Moment of Inertia (SMOI) values. Species names and corresponding specimen number are given above each image.

**
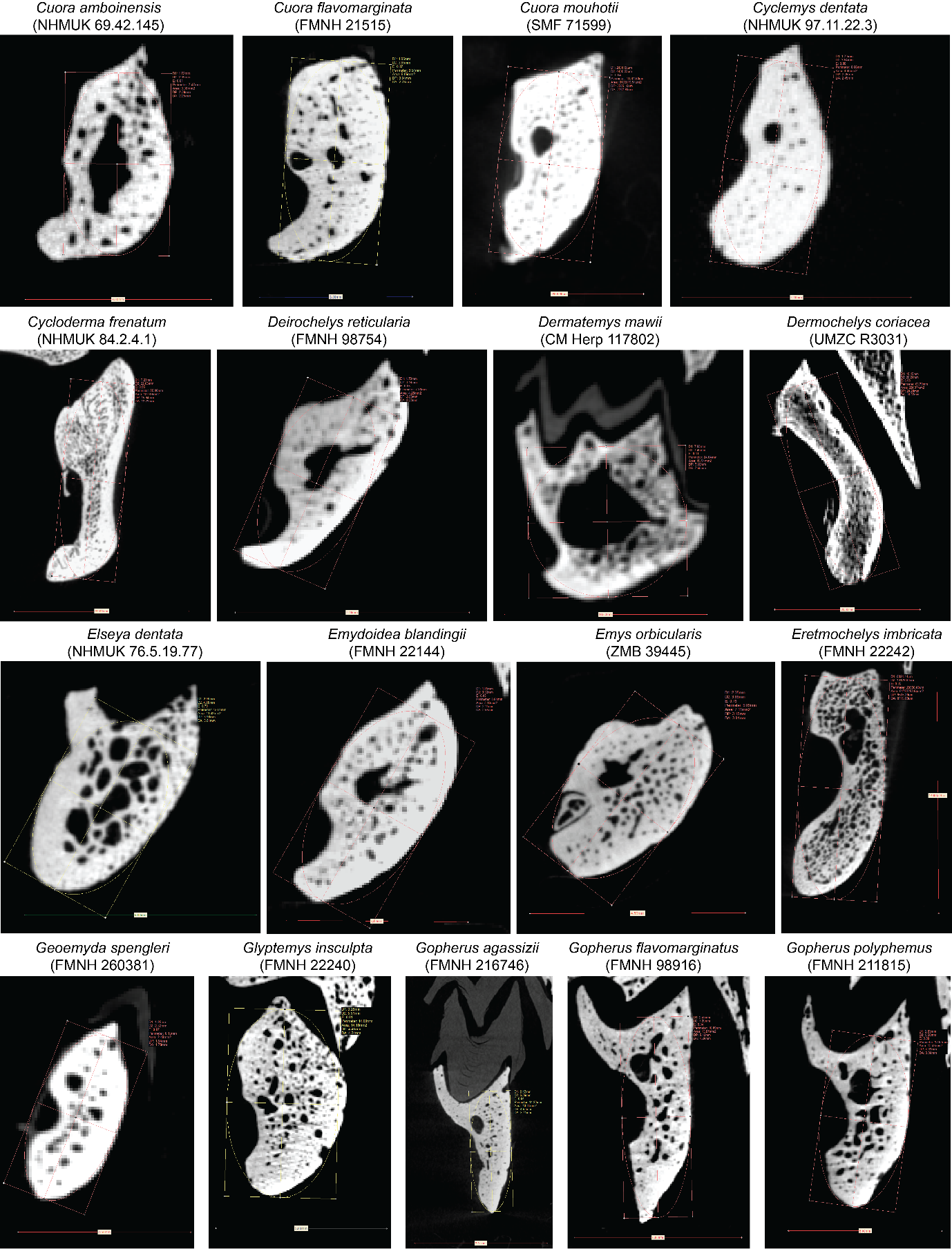
**

**Supplementary Figure S2. Turtle jaw cross-sections (continued).** Cross-sections at the mandibular ramus (see Main Text) used to fit the ellipses to extract Second Moment of Inertia (SMOI) values. Species names and corresponding specimen number are given above each image.


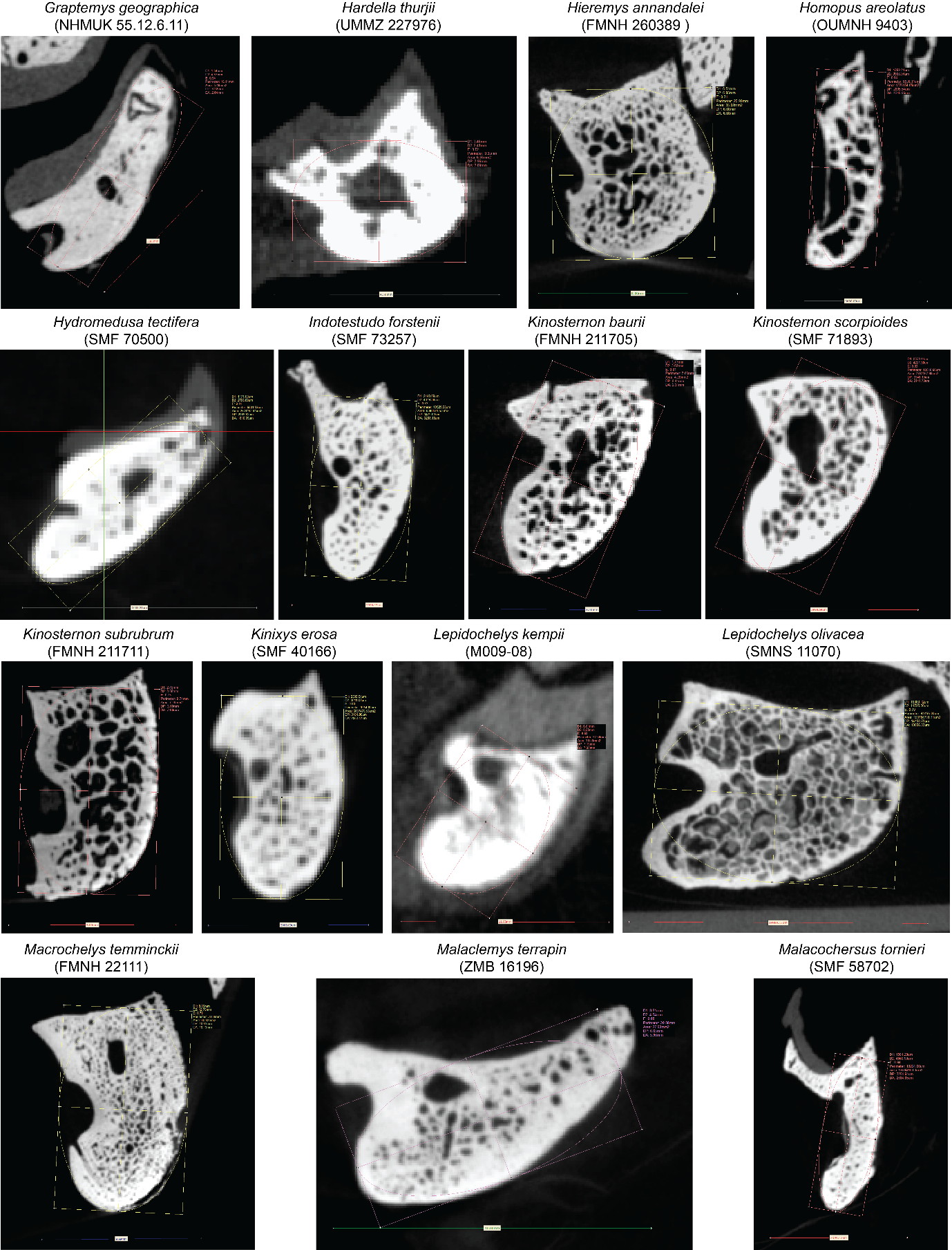


**Supplementary Figure S3. Turtle jaw cross-sections (continued).** Cross-sections at the mandibular ramus (see Main Text) used to fit the ellipses to extract Second Moment of Inertia (SMOI) values. Species names and corresponding specimen number are given above each image.


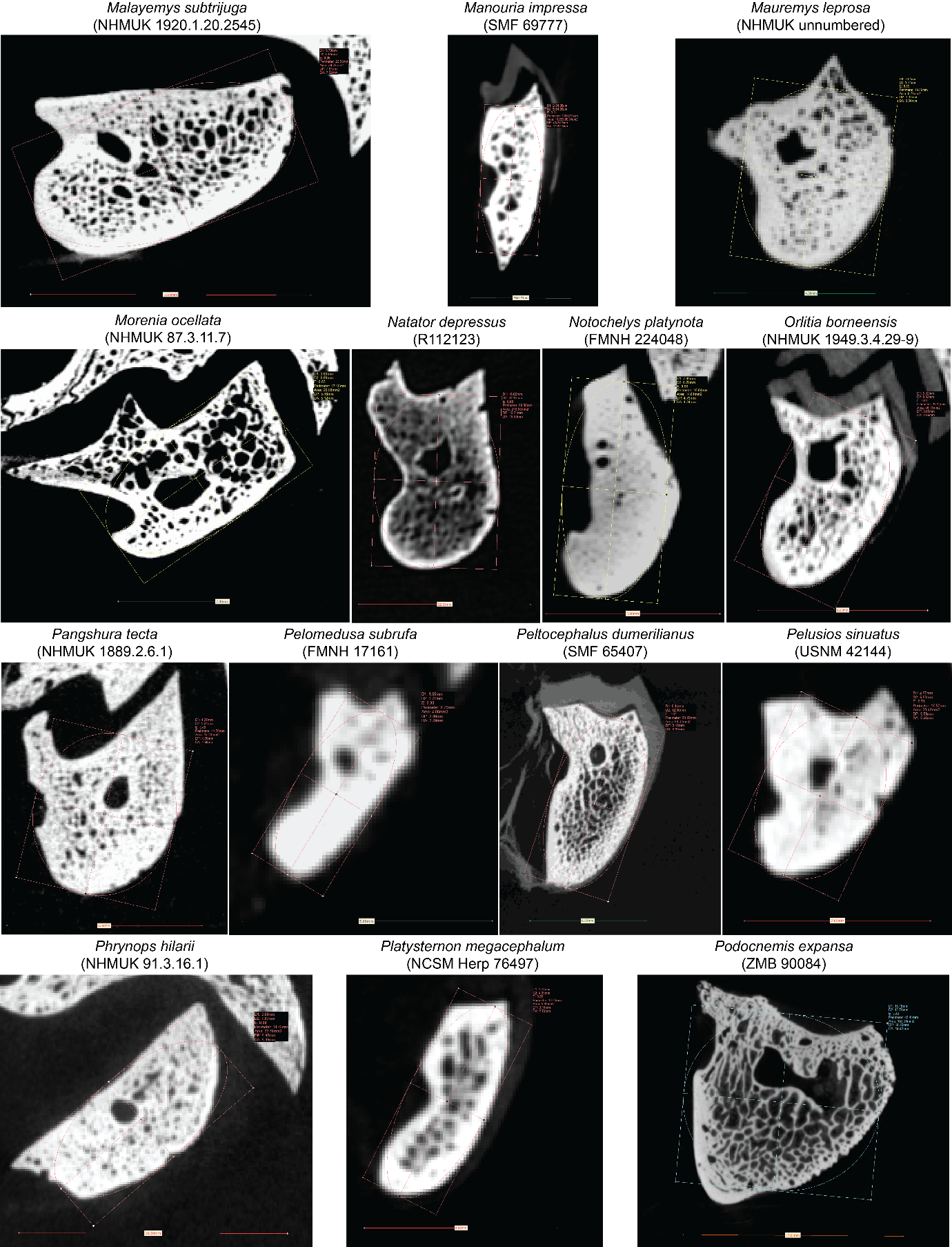


**Supplementary Figure S4. Turtle jaw cross-sections (continued).** Cross-sections at the mandibular ramus (see Main Text) used to fit the ellipses to extract Second Moment of Inertia (SMOI) values. Species names and corresponding specimen number are given above each image.


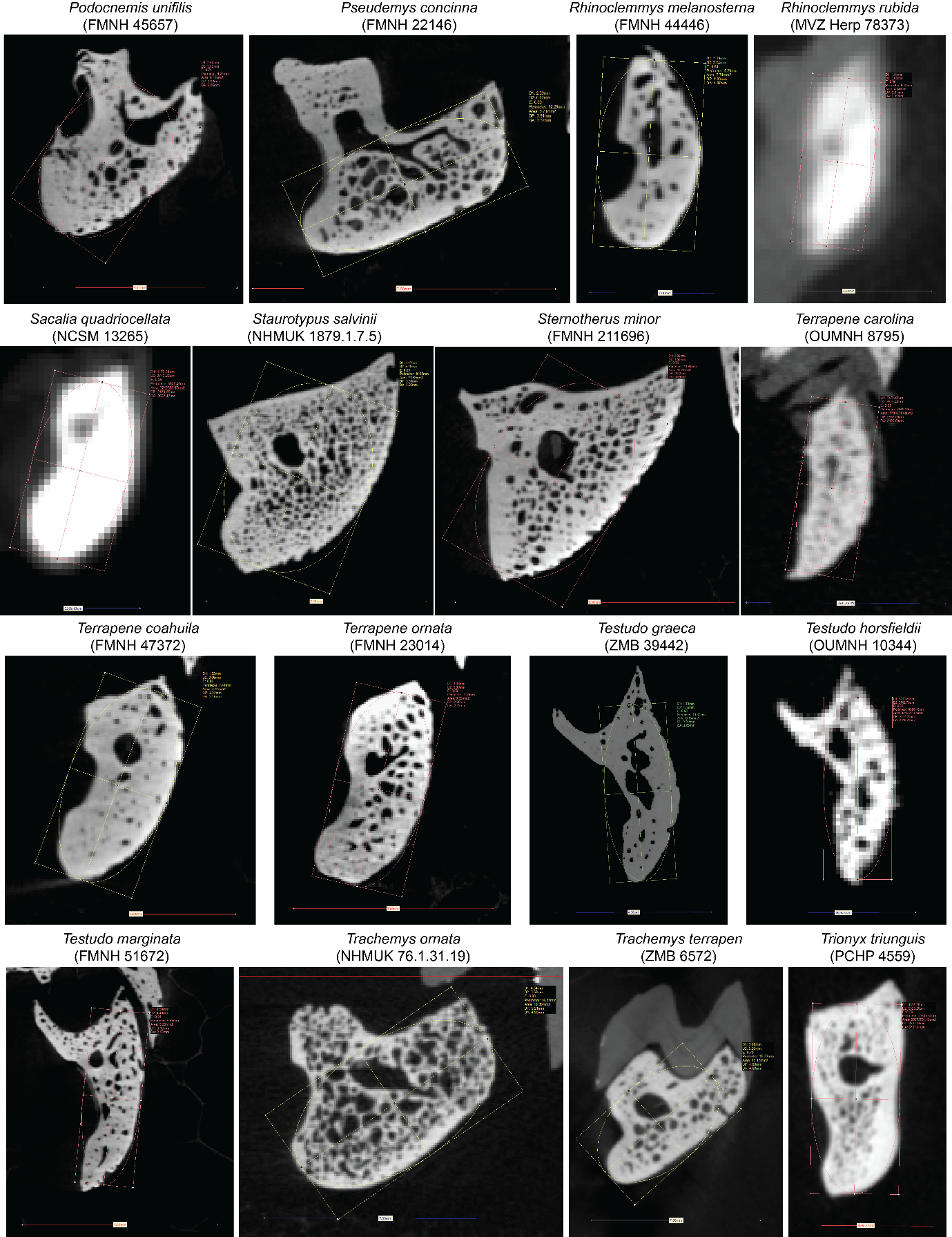


**Supplementary Figure S5. Turtle jaw cross-sections (continued).** Cross-sections at the mandibular ramus (see Main Text) used to fit the ellipses to extract Second Moment of Inertia (SMOI) values. Species names and corresponding specimen number are given above each image.


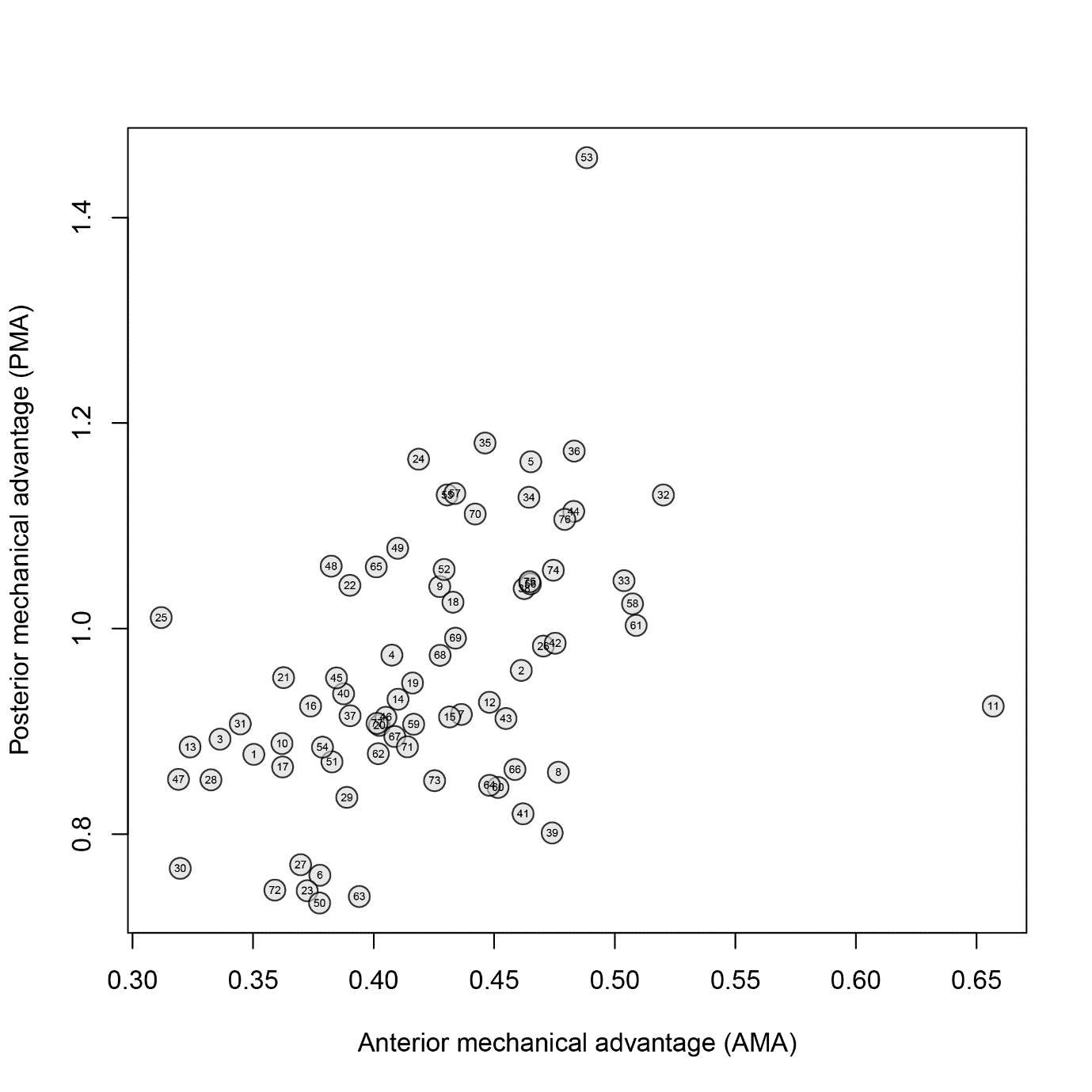


**Supplementary Figure S6.** Correlation between anterior (AMA) and posterior (PMA) mechanical advantages of turtle mandibles (Pearson’s r = 0.44; p = 6.7·10^-5^). Numbers correspond to: 1- *Aldabrachelys gigantea*, 2- *Amyda cartilaginea*, 3- *Apalone mutica*, 4- *Apalone spinifera*, 5- *Batagur baska*, 6- *Caretta caretta*, 7- *Carettochelys insculpta*, 8- *Chelodina oblonga*, 9- *Chelonia mydas*, 10- *Chelonoidis niger*, 11- *Chelus fimbriatus*, 12- *Chelydra serpentina*, 13- *Chitra chitra*, 14- *Chrysemys picta*, 15- *Trachemys terrapen*, 16- *Claudius angustatus*, 17- *Clemmys guttata*, 18- *Cuora amboinensis*, 19- *Cuora flavomarginata*, 20- *Cuora mouhotii*, 21- *Cyclemys dentata*, 22- *Cycloderma frenatum*, 23- *Deirochelys reticularia*, 24- *Dermatemys mawii*, 25- *Dermochelys coriacea*, 26- *Elseya dentata*, 27- *Emydoidea blandingii*, 28- *Emys orbicularis*, 29- *Eretmochelys imbricata*, 30- *Geoemyda spengleri*, 31- *Glyptemys insculpta*, 32- *Gopherus agassizii*, 33- *Gopherus flavomarginatus*, 34- *Gopherus polyphemus*, 35- *Graptemys geographica*, 36- *Hardella thurjii*, 37- *Heosemys annandalii*, 38- *Homopus areolatus*, 39- *Hydromedusa tectifera*, 40- *Indotestudo elongata*, 41- *Kinixys erosa*, 42- *Kinosternon baurii*, 43- *Kinosternon scorpioides*, 44- *Kinosternon subrubrum*, 45- *Lepidochelys kempii*, 46- *Lepidochelys olivacea*, 47- *Macrochelys temminckii*, 48- *Malaclemys terrapin*, 49- *Malacochersus tornieri*, 50- *Malayemys subtrijuga*, 51- *Manouria impressa*, 52- *Mauremys leprosa*, 53- *Morenia ocellata*, 54- *Natator depressus*, 55- *Notochelys platynota*, 56- *Orlitia borneensis*, 57- *Pangshura tecta*, 58- *Pelomedusa subrufa*, 59- *Peltocephalus dumerilianus*, 60- *Pelusios sinuatus*, 61- *Phrynops hilarii*, 62- *Platysternon megacephalum*, 63- *Podocnemis expansa*, 64- *Podocnemis unifilis*, 65- *Pseudemys concinna*, 66- *Rhinoclemmys melanosterna*, 67- *Rhinoclemmys rubida*, 68- *Sacalia quadriocellata*, 69- *Staurotypus salvinii*, 70- *Sternotherus minor*, 71- *Terrapene carolina*, 72- *Terrapene coahuila*, 73- *Terrapene ornata*, 74- *Testudo graeca*, 75- *Testudo horsfieldii*, 76- *Testudo marginata*, 77- *Trionyx triunguis*.


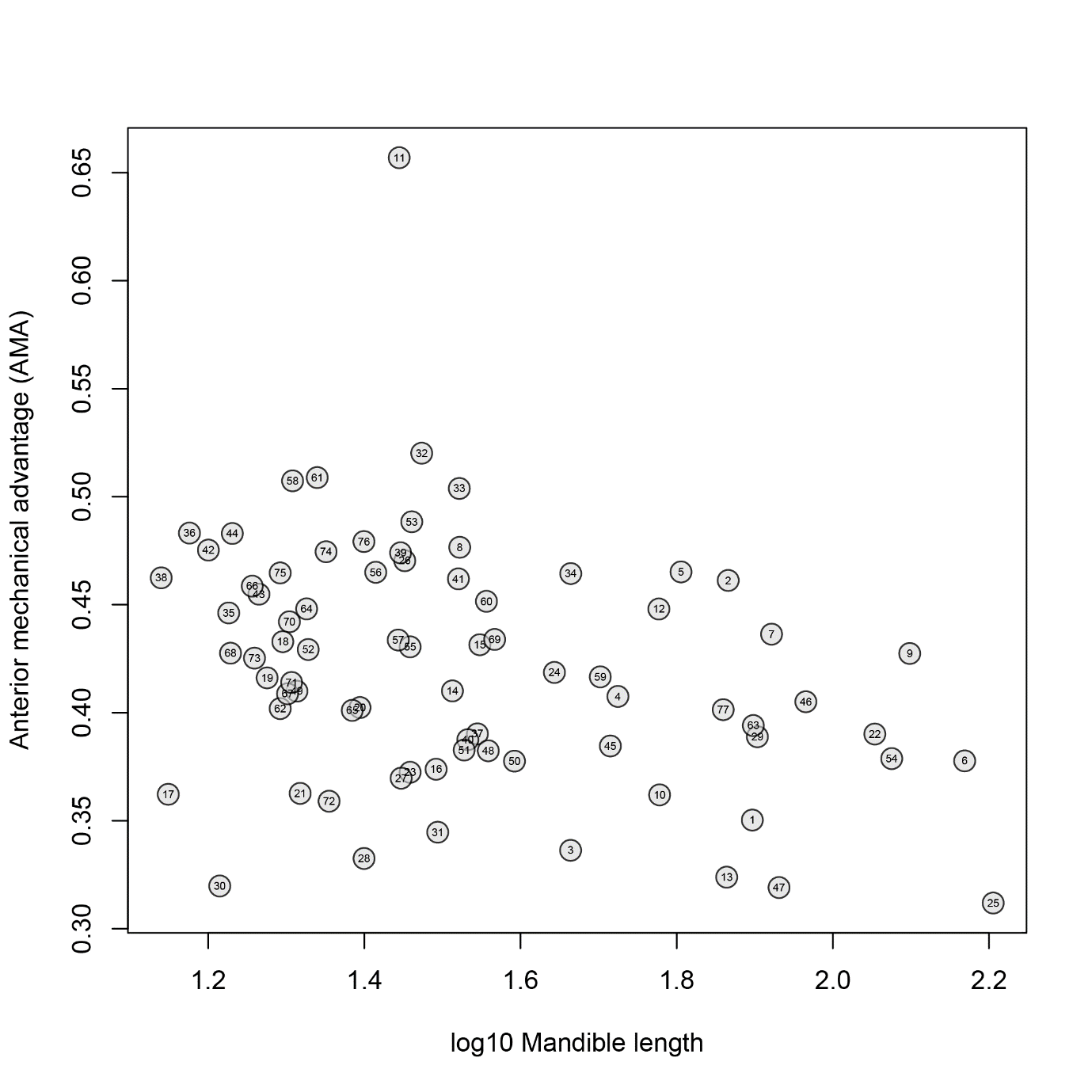


**Supplementary Figure S7.** Correlation between anterior mechanical advantage (AMA) and log_10_-transformed turtle mandible length (Pearson’s r = -0.33; p = 0.003). Numbers correspond to: 1- *Aldabrachelys gigantea*, 2- *Amyda cartilaginea*, 3- *Apalone mutica*, 4- *Apalone spinifera*, 5- *Batagur baska*, 6- *Caretta caretta*, 7- *Carettochelys insculpta*, 8- *Chelodina oblonga*, 9- *Chelonia mydas*, 10- *Chelonoidis niger*, 11- *Chelus fimbriatus*, 12- *Chelydra serpentina*, 13- *Chitra chitra*, 14- *Chrysemys picta*, 15- *Trachemys terrapen*, 16- *Claudius angustatus*, 17- *Clemmys guttata*, 18- *Cuora amboinensis*, 19- *Cuora flavomarginata*, 20- *Cuora mouhotii*, 21- *Cyclemys dentata*, 22- *Cycloderma frenatum*, 23- *Deirochelys reticularia*, 24- *Dermatemys mawii*, 25- *Dermochelys coriacea*, 26- *Elseya dentata*, 27- *Emydoidea blandingii*, 28- *Emys orbicularis*, 29- *Eretmochelys imbricata*, 30- *Geoemyda spengleri*, 31- *Glyptemys insculpta*, 32- *Gopherus agassizii*, 33- *Gopherus flavomarginatus*, 34- *Gopherus polyphemus*, 35- *Graptemys geographica*, 36- *Hardella thurjii*, 37- *Heosemys annandalii*, 38- *Homopus areolatus*, 39- *Hydromedusa tectifera*, 40- *Indotestudo elongata*, 41- *Kinixys erosa*, 42- *Kinosternon baurii*, 43- *Kinosternon scorpioides*, 44- *Kinosternon subrubrum*, 45- *Lepidochelys kempii*, 46- *Lepidochelys olivacea*, 47- *Macrochelys temminckii*, 48- *Malaclemys terrapin*, 49- *Malacochersus tornieri*, 50- *Malayemys subtrijuga*, 51- *Manouria impressa*, 52- *Mauremys leprosa*, 53- *Morenia ocellata*, 54- *Natator depressus*, 55- *Notochelys platynota*, 56- *Orlitia borneensis*, 57- *Pangshura tecta*, 58- *Pelomedusa subrufa*, 59- *Peltocephalus dumerilianus*, 60- *Pelusios sinuatus*, 61- *Phrynops hilarii*, 62- *Platysternon megacephalum*, 63- *Podocnemis expansa*, 64- *Podocnemis unifilis*, 65- *Pseudemys concinna*, 66- *Rhinoclemmys melanosterna*, 67- *Rhinoclemmys rubida*, 68- *Sacalia quadriocellata*, 69- *Staurotypus salvinii*, 70- *Sternotherus minor*, 71- *Terrapene carolina*, 72- *Terrapene coahuila*, 73- *Terrapene ornata*, 74- *Testudo graeca*, 75- *Testudo horsfieldii*, 76- *Testudo marginata*, 77- *Trionyx triunguis*.


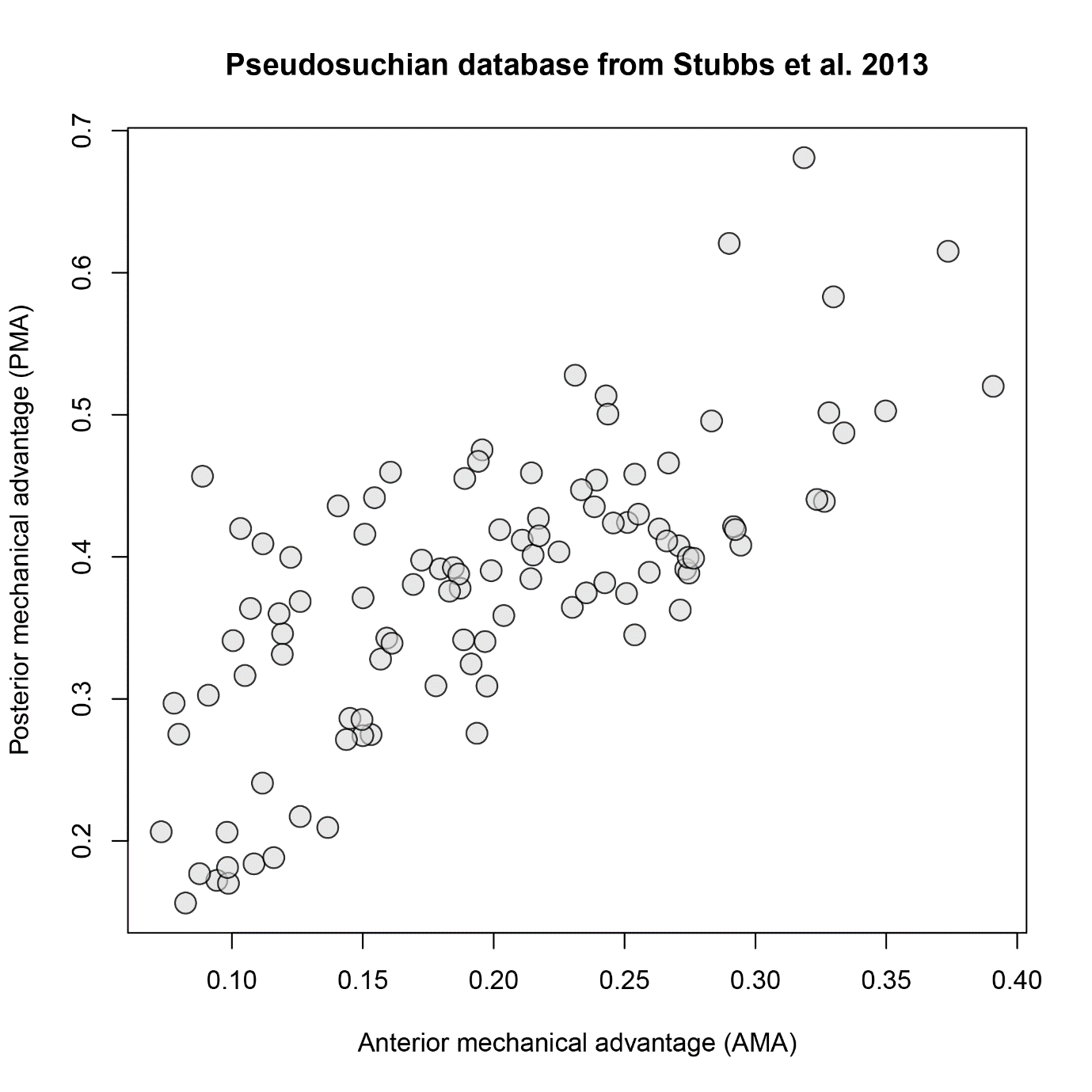


**Supplementary Figure S8.** Correlation between anterior (AMA) and posterior (PMA) mechanical advantages of pseudosuchian mandibles, extracted from the dataset of Stubbs et al. (2013: ‘*Morphological and biomechanical disparity of crocodile-line archosaurs following the end-Triassic extinction*’, Proc Royal Soc B, 280, 20131940, <https://doi.org/10.1098/rspb.2013.1940>). Different from turtles, this correlation is stronger for pseudosuchians (Pearson’s r = 0.72, p = 2.2·10^-16^).


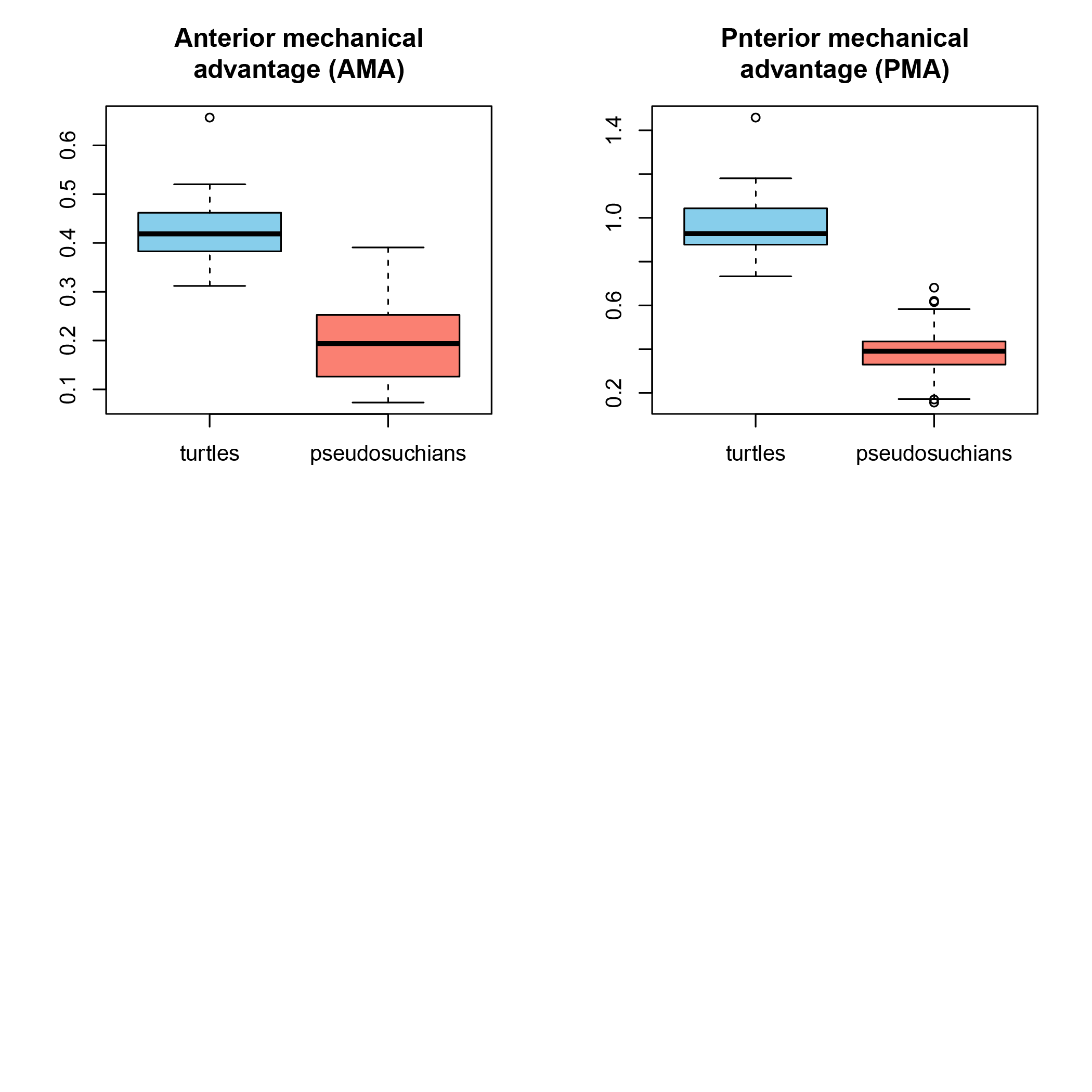


**Supplementary Figure S9.** Boxplots comparing anterior (AMA; left) and posterior (PMA; right) mechanical advantages of turtle and pseudosuchian mandibles. The pseudosuchian data was extracted from the dataset of Stubbs et al. (2013; see above for reference). T-tests show that both groups are statistically different from one another (see Main Text for numerical results).


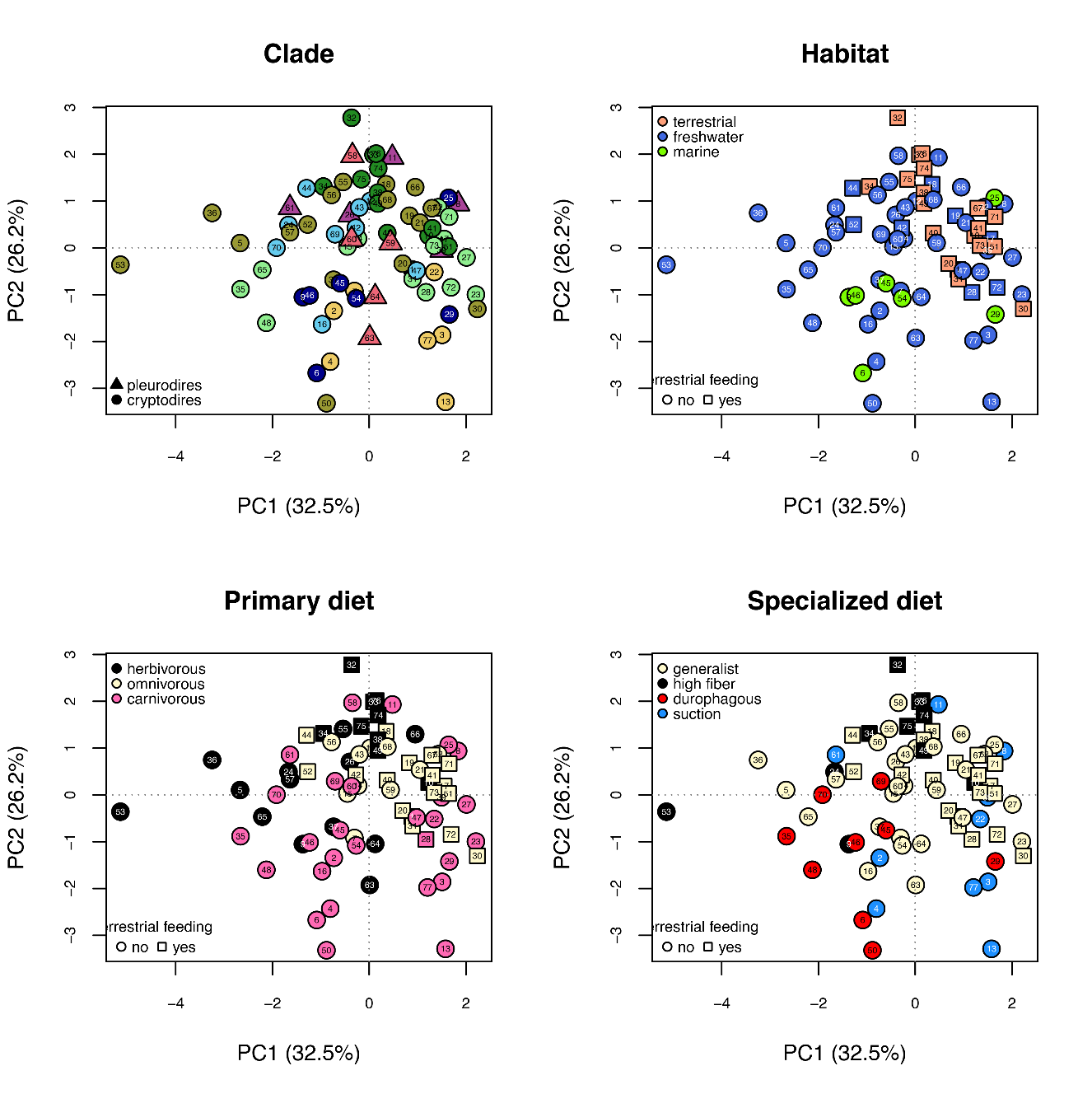


**Supplementary Figure S10.** PCA morphospace of functional jaw measurements based on first two principal components, with various grouping superimposed on the data. Refer to Main Text for clade colour caption in panel (A). Numbers in the panels correspond to: 1- *Aldabrachelys gigantea*, 2- *Amyda cartilaginea*, 3- *Apalone mutica*, 4- *Apalone spinifera*, 5- *Batagur baska*, 6- *Caretta caretta*, 7- *Carettochelys insculpta*, 8- *Chelodina oblonga*, 9- *Chelonia mydas*, 10- *Chelonoidis niger*, 11- *Chelus fimbriatus*, 12- *Chelydra serpentina*, 13- *Chitra chitra*, 14- *Chrysemys picta*, 15- *Trachemys terrapen*, 16- *Claudius angustatus*, 17- *Clemmys guttata*, 18- *Cuora amboinensis*, 19- *Cuora flavomarginata*, 20- *Cuora mouhotii*, 21- *Cyclemys dentata*, 22- *Cycloderma frenatum*, 23- *Deirochelys reticularia*, 24- *Dermatemys mawii*, 25- *Dermochelys coriacea*, 26- *Elseya dentata*, 27- *Emydoidea blandingii*, 28- *Emys orbicularis*, 29- *Eretmochelys imbricata*, 30- *Geoemyda spengleri*, 31- *Glyptemys insculpta*, 32- *Gopherus agassizii*, 33- *Gopherus flavomarginatus*, 34- *Gopherus polyphemus*, 35- *Graptemys geographica*, 36- *Hardella thurjii*, 37- *Heosemys annandalii*, 38- *Homopus areolatus*, 39- *Hydromedusa tectifera*, 40- *Indotestudo elongata*, 41- *Kinixys erosa*, 42- *Kinosternon baurii*, 43- *Kinosternon scorpioides*, 44- *Kinosternon subrubrum*, 45- *Lepidochelys kempii*, 46- *Lepidochelys olivacea*, 47- *Macrochelys temminckii*, 48- *Malaclemys terrapin*, 49- *Malacochersus tornieri*, 50- *Malayemys subtrijuga*, 51- *Manouria impressa*, 52- *Mauremys leprosa*, 53- *Morenia ocellata*, 54- *Natator depressus*, 55- *Notochelys platynota*, 56- *Orlitia borneensis*, 57- *Pangshura tecta*, 58- *Pelomedusa subrufa*, 59- *Peltocephalus dumerilianus*, 60- *Pelusios sinuatus*, 61- *Phrynops hilarii*, 62- *Platysternon megacephalum*, 63- *Podocnemis expansa*, 64- *Podocnemis unifilis*, 65- *Pseudemys concinna*, 66- *Rhinoclemmys melanosterna*, 67- *Rhinoclemmys rubida*, 68- *Sacalia quadriocellata*, 69- *Staurotypus salvinii*, 70- *Sternotherus minor*, 71- *Terrapene carolina*, 72- *Terrapene coahuila*, 73- *Terrapene ornata*, 74- *Testudo graeca*, 75- *Testudo horsfieldii*, 76- *Testudo marginata*, 77- *Trionyx triunguis*.

**
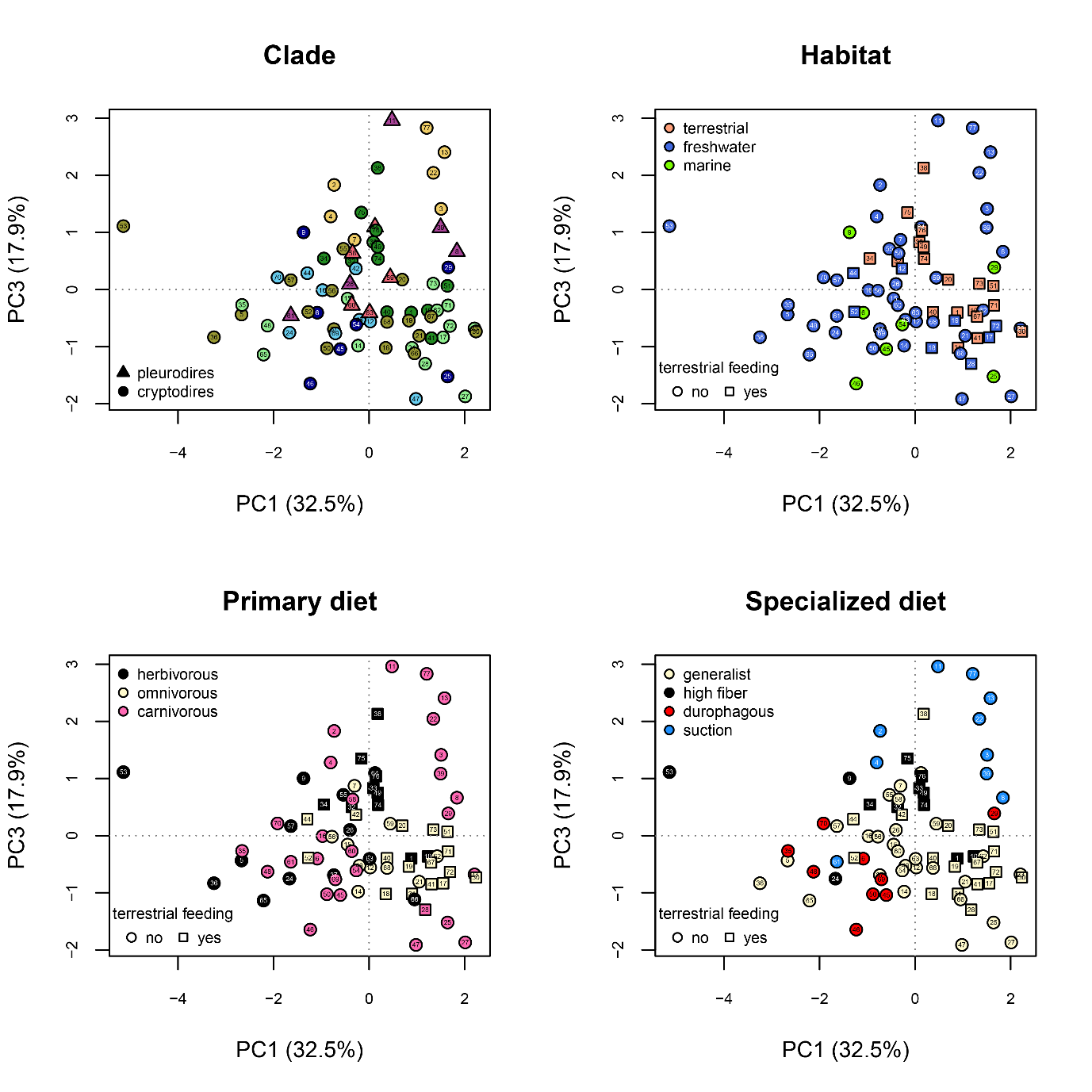
**

**Supplementary Figure S11.** PCA morphospace of functional jaw measurements based on first and third principal components, with various grouping superimposed on the data. Refer to Main Text for clade colour caption in panel (A). Numbers in the panels correspond to: 1- *Aldabrachelys gigantea*, 2- *Amyda cartilaginea*, 3- *Apalone mutica*, 4- *Apalone spinifera*, 5- *Batagur baska*, 6- *Caretta caretta*, 7- *Carettochelys insculpta*, 8- *Chelodina oblonga*, 9- *Chelonia mydas*, 10- *Chelonoidis niger*, 11- *Chelus fimbriatus*, 12- *Chelydra serpentina*, 13- *Chitra chitra*, 14- *Chrysemys picta*, 15- *Trachemys terrapen*, 16- *Claudius angustatus*, 17- *Clemmys guttata*, 18- *Cuora amboinensis*, 19- *Cuora flavomarginata*, 20- *Cuora mouhotii*, 21- *Cyclemys dentata*, 22- *Cycloderma frenatum*, 23- *Deirochelys reticularia*, 24- *Dermatemys mawii*, 25- *Dermochelys coriacea*, 26- *Elseya dentata*, 27- *Emydoidea blandingii*, 28- *Emys orbicularis*, 29- *Eretmochelys imbricata*, 30- *Geoemyda spengleri*, 31- *Glyptemys insculpta*, 32- *Gopherus agassizii*, 33- *Gopherus flavomarginatus*, 34- *Gopherus polyphemus*, 35- *Graptemys geographica*, 36- *Hardella thurjii*, 37- *Heosemys annandalii*, 38- *Homopus areolatus*, 39- *Hydromedusa tectifera*, 40- *Indotestudo elongata*, 41- *Kinixys erosa*, 42- *Kinosternon baurii*, 43- *Kinosternon scorpioides*, 44- *Kinosternon subrubrum*, 45- *Lepidochelys kempii*, 46- *Lepidochelys olivacea*, 47- *Macrochelys temminckii*, 48- *Malaclemys terrapin*, 49- *Malacochersus tornieri*, 50- *Malayemys subtrijuga*, 51- *Manouria impressa*, 52- *Mauremys leprosa*, 53- *Morenia ocellata*, 54- *Natator depressus*, 55- *Notochelys platynota*, 56- *Orlitia borneensis*, 57- *Pangshura tecta*, 58- *Pelomedusa subrufa*, 59- *Peltocephalus dumerilianus*, 60- *Pelusios sinuatus*, 61- *Phrynops hilarii*, 62- *Platysternon megacephalum*, 63- *Podocnemis expansa*, 64- *Podocnemis unifilis*, 65- *Pseudemys concinna*, 66- *Rhinoclemmys melanosterna*, 67- *Rhinoclemmys rubida*, 68- *Sacalia quadriocellata*, 69- *Staurotypus salvinii*, 70- *Sternotherus minor*, 71- *Terrapene carolina*, 72- *Terrapene coahuila*, 73- *Terrapene ornata*, 74- *Testudo graeca*, 75- *Testudo horsfieldii*, 76- *Testudo marginata*, 77- *Trionyx triunguis*.

**
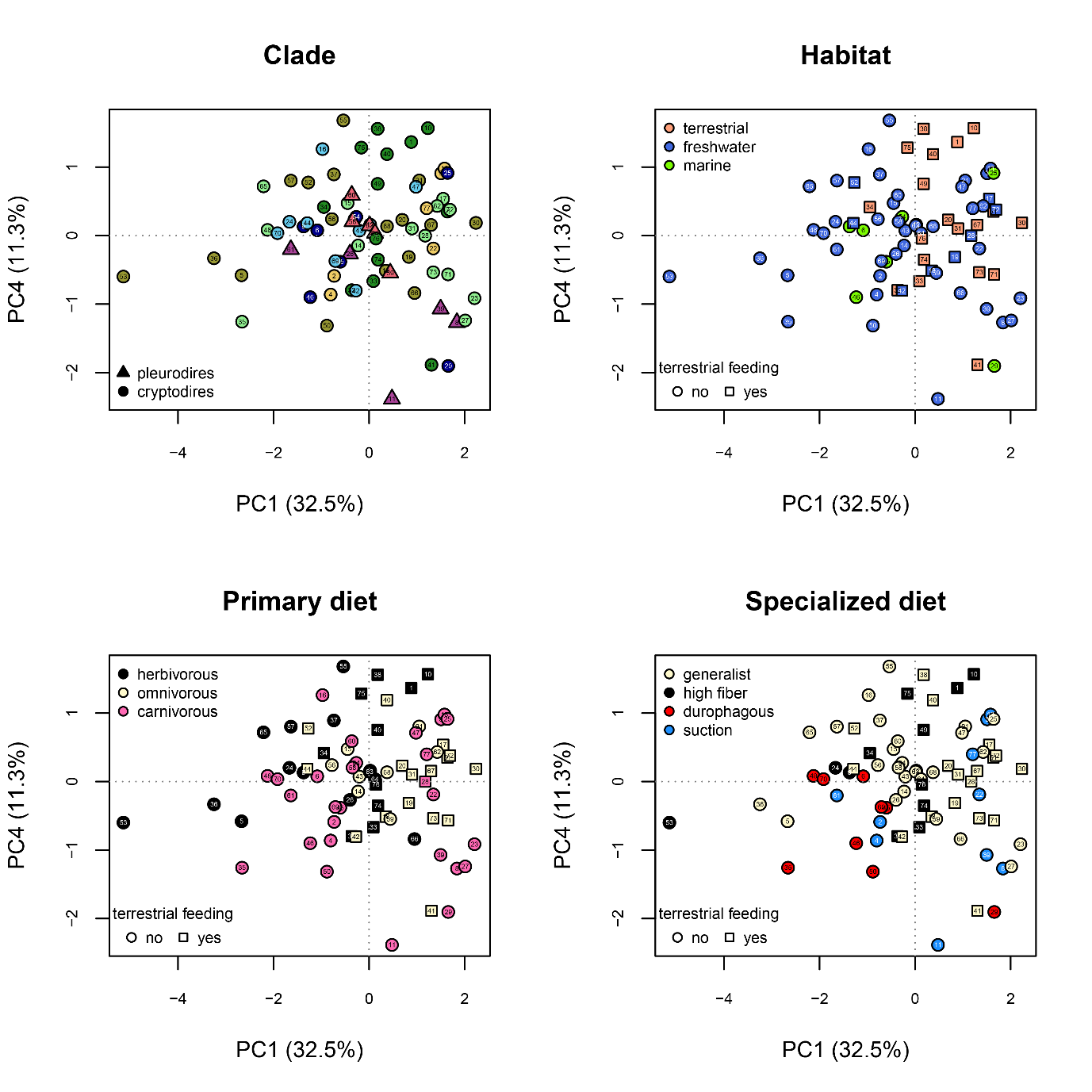
**

**Supplementary Figure S12.** PCA morphospace of functional jaw measurements based on first and fourth principal components, with various grouping superimposed on the data. Refer to Main Text for clade colour caption in panel (A). Numbers in the panels correspond to: 1- *Aldabrachelys gigantea*, 2- *Amyda cartilaginea*, 3- *Apalone mutica*, 4- *Apalone spinifera*, 5- *Batagur baska*, 6- *Caretta caretta*, 7- *Carettochelys insculpta*, 8- *Chelodina oblonga*, 9- *Chelonia mydas*, 10- *Chelonoidis niger*, 11- *Chelus fimbriatus*, 12- *Chelydra serpentina*, 13- *Chitra chitra*, 14- *Chrysemys picta*, 15- *Trachemys terrapen*, 16- *Claudius angustatus*, 17- *Clemmys guttata*, 18- *Cuora amboinensis*, 19- *Cuora flavomarginata*, 20- *Cuora mouhotii*, 21- *Cyclemys dentata*, 22- *Cycloderma frenatum*, 23- *Deirochelys reticularia*, 24- *Dermatemys mawii*, 25- *Dermochelys coriacea*, 26- *Elseya dentata*, 27- *Emydoidea blandingii*, 28- *Emys orbicularis*, 29- *Eretmochelys imbricata*, 30- *Geoemyda spengleri*, 31- *Glyptemys insculpta*, 32- *Gopherus agassizii*, 33- *Gopherus flavomarginatus*, 34- *Gopherus polyphemus*, 35- *Graptemys geographica*, 36- *Hardella thurjii*, 37- *Heosemys annandalii*, 38- *Homopus areolatus*, 39- *Hydromedusa tectifera*, 40- *Indotestudo elongata*, 41- *Kinixys erosa*, 42- *Kinosternon baurii*, 43- *Kinosternon scorpioides*, 44- *Kinosternon subrubrum*, 45- *Lepidochelys kempii*, 46- *Lepidochelys olivacea*, 47- *Macrochelys temminckii*, 48- *Malaclemys terrapin*, 49- *Malacochersus tornieri*, 50- *Malayemys subtrijuga*, 51- *Manouria impressa*, 52- *Mauremys leprosa*, 53- *Morenia ocellata*, 54- *Natator depressus*, 55- *Notochelys platynota*, 56- *Orlitia borneensis*, 57- *Pangshura tecta*, 58- *Pelomedusa subrufa*, 59- *Peltocephalus dumerilianus*, 60- *Pelusios sinuatus*, 61- *Phrynops hilarii*, 62- *Platysternon megacephalum*, 63- *Podocnemis expansa*, 64- *Podocnemis unifilis*, 65- *Pseudemys concinna*, 66- *Rhinoclemmys melanosterna*, 67- *Rhinoclemmys rubida*, 68- *Sacalia quadriocellata*, 69- *Staurotypus salvinii*, 70- *Sternotherus minor*, 71- *Terrapene carolina*, 72- *Terrapene coahuila*, 73- *Terrapene ornata*, 74- *Testudo graeca*, 75- *Testudo horsfieldii*, 76- *Testudo marginata*, 77- *Trionyx triunguis*.


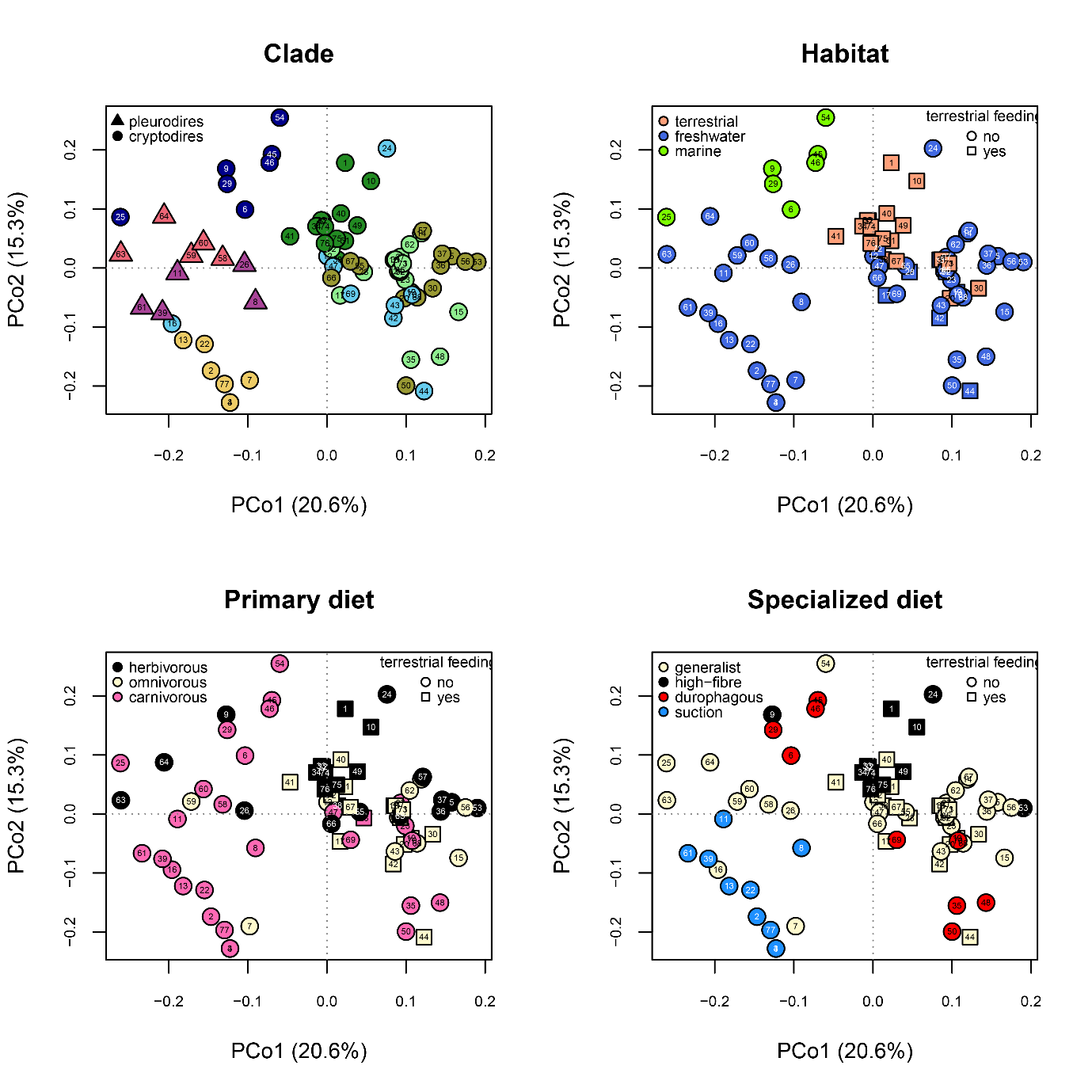


**Supplementary Figure S13.** PCoA morphospace of mandibular character scorings based on first two principal coordinate axes, with various grouping superimposed on the data. Refer to Main Text for clade colour caption in panel (A). Numbers in the panels correspond to: 1- *Aldabrachelys gigantea*, 2- *Amyda cartilaginea*, 3- *Apalone mutica*, 4- *Apalone spinifera*, 5- *Batagur baska*, 6- *Caretta caretta*, 7- *Carettochelys insculpta*, 8- *Chelodina oblonga*, 9- *Chelonia mydas*, 10- *Chelonoidis niger*, 11- *Chelus fimbriatus*, 12- *Chelydra serpentina*, 13- *Chitra chitra*, 14- *Chrysemys picta*, 15- *Trachemys terrapen*, 16- *Claudius angustatus*, 17- *Clemmys guttata*, 18- *Cuora amboinensis*, 19- *Cuora flavomarginata*, 20- *Cuora mouhotii*, 21- *Cyclemys dentata*, 22- *Cycloderma frenatum*, 23- *Deirochelys reticularia*, 24- *Dermatemys mawii*, 25- *Dermochelys coriacea*, 26- *Elseya dentata*, 27- *Emydoidea blandingii*, 28- *Emys orbicularis*, 29- *Eretmochelys imbricata*, 30- *Geoemyda spengleri*, 31- *Glyptemys insculpta*, 32- *Gopherus agassizii*, 33- *Gopherus flavomarginatus*, 34- *Gopherus polyphemus*, 35- *Graptemys geographica*, 36- *Hardella thurjii*, 37- *Heosemys annandalii*, 38- *Homopus areolatus*, 39- *Hydromedusa tectifera*, 40- *Indotestudo elongata*, 41- *Kinixys erosa*, 42- *Kinosternon baurii*, 43- *Kinosternon scorpioides*, 44- *Kinosternon subrubrum*, 45- *Lepidochelys kempii*, 46- *Lepidochelys olivacea*, 47- *Macrochelys temminckii*, 48- *Malaclemys terrapin*, 49- *Malacochersus tornieri*, 50- *Malayemys subtrijuga*, 51- *Manouria impressa*, 52- *Mauremys leprosa*, 53- *Morenia ocellata*, 54- *Natator depressus*, 55- *Notochelys platynota*, 56- *Orlitia borneensis*, 57- *Pangshura tecta*, 58- *Pelomedusa subrufa*, 59- *Peltocephalus dumerilianus*, 60- *Pelusios sinuatus*, 61- *Phrynops hilarii*, 62- *Platysternon megacephalum*, 63- *Podocnemis expansa*, 64- *Podocnemis unifilis*, 65- *Pseudemys concinna*, 66- *Rhinoclemmys melanosterna*, 67- *Rhinoclemmys rubida*, 68- *Sacalia quadriocellata*, 69- *Staurotypus salvinii*, 70- *Sternotherus minor*, 71- *Terrapene carolina*, 72- *Terrapene coahuila*, 73- *Terrapene ornata*, 74- *Testudo graeca*, 75- *Testudo horsfieldii*, 76- *Testudo marginata*, 77- *Trionyx triunguis*.

**Supplementary Table S1.** Variance explained by each axis of the PCoA analysis and its respective importance (cumulative variance). The first 18 axes explain around 95% of the total variance.

|  | **variance (%)** | **cumulative variance (%)** |
| --- | --- | --- |
| **Axis 1** | 20.6 | 20.6 |
| **Axis 2** | 15.25 | 35.86 |
| **Axis 3** | 9.69 | 45.55 |
| **Axis 4** | 8.93 | 54.47 |
| **Axis 5** | 6.66 | 61.14 |
| **Axis 6** | 6.39 | 67.53 |
| **Axis 7** | 5.36 | 72.89 |
| **Axis 8** | 4.03 | 76.92 |
| **Axis 9** | 3.19 | 80.1 |
| **Axis 10** | 2.84 | 82.94 |
| **Axis 11** | 2.34 | 85.28 |
| **Axis 12** | 2.16 | 87.44 |
| **Axis 13** | 1.93 | 89.37 |
| **Axis 14** | 1.81 | 91.18 |
| **Axis 15** | 1.47 | 92.65 |
| **Axis 16** | 1.21 | 93.85 |
| **Axis 17** | 1.06 | 94.91 |
| **Axis 18** | 1.03 | 95.94 |
| **Axis 19** | 0.88 | 96.82 |
| **Axis 20** | 0.6 | 97.43 |
| **Axis 21** | 0.53 | 97.96 |
| **Axis 22** | 0.47 | 98.43 |
| **Axis 23** | 0.42 | 98.85 |
| **Axis 24** | 0.32 | 99.17 |
| **Axis 25** | 0.26 | 99.43 |
| **Axis 26** | 0.22 | 99.65 |
| **Axis 27** | 0.16 | 99.82 |
| **Axis 28** | 0.11 | 99.93 |
| **Axis 29** | 0.04 | 99.97 |
| **Axis 30** | 0.03 | 100 |
